# Green solvents, materials, and lead-free semiconductors for sustainable fabrication of perovskite solar cells

**DOI:** 10.1039/d3ra01692g

**Published:** 2023-06-15

**Authors:** Suresh K. Podapangi, Farshad Jafarzadeh, Sara Mattiello, Tulja Bhavani Korukonda, Akash Singh, Luca Beverina, Thomas M. Brown

**Affiliations:** a CHOSE (Centre for Hybrid and Organic Solar Energy), Department of Electronic Engineering, University of Rome-Tor Vergata via del Politecnico 1 00133 Rome Italy Thomas.brown@uniroma2.it; b Department of Materials Science, State University of Milano-Bicocca Via Cozzi 55 I-20126 Milano Italy; c Department of Centre for Energy Studies, Indian Institute of Technology Delhi Hauz Khas New Delhi-110016 India; d Department of Mechanical Engineering and Materials Science, Duke University Durham NC 27708 USA

## Abstract

Perovskite materials research has received unprecedented recognition due to its applications in photovoltaics, LEDs, and other large area low-cost electronics. The exceptional improvement in the photovoltaic conversion efficiency of Perovskite solar cells (PSCs) achieved over the last decade has prompted efforts to develop and optimize device fabrication technologies for the industrial and commercial space. However, unstable operation in outdoor environments and toxicity of the employed materials and solvents have hindered this proposition. While their optoelectronic properties are extensively studied, the environmental impacts of the materials and manufacturing methods require further attention. This review summarizes and discusses green and environment-friendly methods for fabricating PSCs, particularly non-toxic solvents, and lead-free alternatives. Greener solvent choices are surveyed for all the solar cell films, (*i.e.* electron and hole transport, semiconductor, and electrode layers) and their impact on thin film quality, morphology and device performance is explored. We also discuss lead content in perovskites, its environmental impact and sequestration routes, and progress in replacing lead with greener alternatives. This review provides an analysis of sustainable green routes in perovskite solar cell fabrication, discussing the impact of each layer in the device stack, *via* life cycle analysis.

## Abstract 2

This review discusses the use of green and environment-friendly methods for fabricating perovskite solar cells (PSCs), including non-toxic solvents and lead-free alternatives.

## Graphical abstract

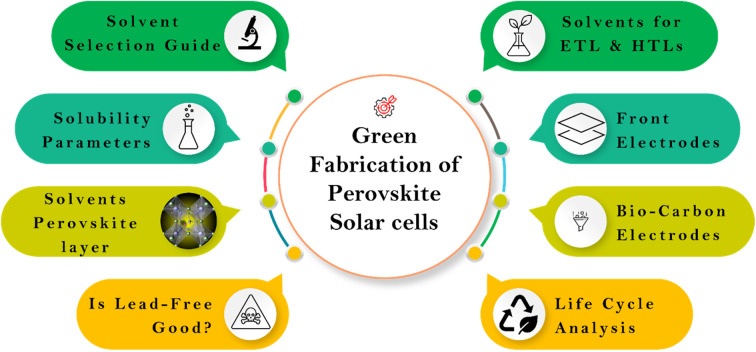

## 1. Introduction

Metal Halide Perovskites (MHPs) have revolutionized the field of optoelectronics and garnered worldwide interest due to their outstanding semiconducting characteristics complemented by structure–property tunability and facile deposition techniques.^1^ Such attributes have made perovskite a ubiquitous material in the research domain with potential applications in fields of photovoltaics, light-emitting diodes, lasers, data storage and computing devices. PSCs have experienced an unparalleled swift rise in photovoltaic power conversion efficiency (PCE) from 3.8% to 25.7% in just over a decade^2–4^ surpassing other emerging photovoltaic technologies.^4^ High absorption coefficients over broad spectral ranges, defect tolerance to deep gap states, low exciton binding energy^5^ and excellent ambipolar charge transport^6^ are salient features of perovskites that make them suited for solar devices. In general, perovskites have structural framework of multiple dimensionality (3D, 2D, quasi-2D, 1D and 0D).^7^ However, the most studied systems are 3D and 2D with ABX_3_ and A′_2_BX_4_ compositions respectively where, A is a monovalent small cation, A′ is a bigger alkyl or aryl ammonium cation, B a divalent metal cation and X a halogen.^8–10^

Although perovskite PV has great potential, research and development has major challenges to tackle before commercialization, mainly stability under operating conditions, and developing economic and scalable fabrication process that can be industrialized. In addition, it is necessary to make sure both the end product and the manufacturing process are environmentally friendly and with a low carbon footprint answering at the same time regulatory and environmental concerns. Nowadays, PSCs encounter two critical issues that are of the utmost importance. Primarily, the presence of lead in the photoactive semiconductor, and secondly the toxicity of solvents used in fabrication. Both need to be addressed. Attempts have been directed to replace the 3-D framework of lead with periodic table group elements 14 and 15, or transition metals.^11,12^ The incorporation of these alternative elements has come with a lowering in device stability and efficiency, as discussed in detail in Section 4. The toxicity of the solvents used in the processing of active and charge transport layers is the other and less frequently discussed concern. In large scale manufacturing, PSC possesses the advantage of deposition by solution processing. However, the majority of current methods use toxic solvents like *N*,*N*-dimethylformamide (DMF) and *N*-methylpyrrolidone (NMP) for perovskite deposition^13^ and chlorobenzene (CB), di-chlorobenzene (DCB), for hole transport materials such as spiro-OMeTAD and poly(triarylamine) (PTAA).^14–16^ To develop alternative green solvents three key factors to be considered are: (i) solute–solvent interactions *i.e.*, solubility of the precursors, (ii) solution–substrate interactions *i.e.*, wettability of the substrate and (iii) formation of high-quality conformal layers.^17^ Further, the deposition of individual layers in a complete device should not interfere with the quality of the already-deposited underlying layer for proper stacking of layers.

Recently, several articles have discussed recent advancements in the production of greener perovskite solar cells (PSCs).^18–21^ These advancements include the use and effects of solvents and antisolvents that can be classified as eco-friendly on perovskite synthesis, film quality and device performance, the potential environmental and health hazards from materials even for mass production, the sustainability and lead handling and recycling technologies. This review focuses on summarizing current progress in developing more environmentally-friendly perovskite solar cells from many angles. It not only covers recent advances in green fabrication, including the use of green solvents for the perovskite layer. It also provides a detailed description for each layer of the device including those for transport, and front, and back contacts including life cycle assessment considerations as well as lead free perovskite alternatives.

**Fig. 1:**
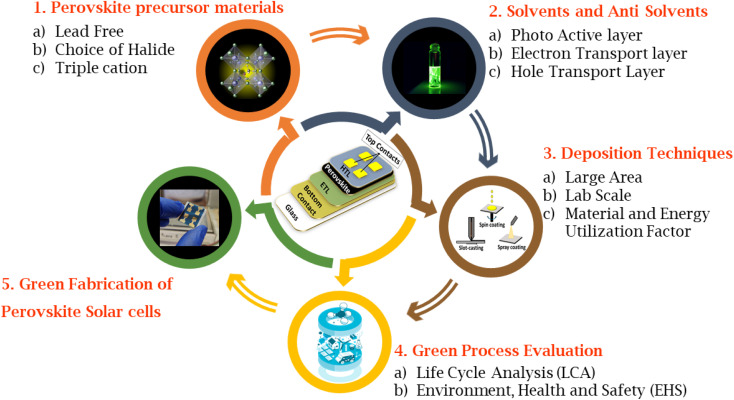
Schematic diagram representing the different stages of green fabrication of PSCs system that include: (1) perovskite precursor materials, (2) solvents and anti-solvents, (3) deposition techniques, (4) green process evaluation, (5) complete green fabrication of PSCs.

Fig. 1 summarizes different stages of green fabrication of PSCs system that include: (1) perovskite precursor materials, (2) solvents and anti-solvents, (3) deposition techniques, (4) green process evaluation, (5) complete green fabrication of PSCs. In this review, we focus on the environmentally friendly sustainable fabrication techniques and replacement of toxic elements of PSCs. Section 2 deals with the selection and description of green solvents. Sections 3 provides a comprehensive discussion of alternative solvents for replacing toxic solvents in the fabrication of PSCs. Section 4 supplies a focused literature survey on lead-free perovskites. We further explore alternative charge transport layers (Section 5), electrode materials (Section 6) and green solvents that can be used in their deposition process in dedicated subsections. Finally, an overview of life cycle analysis (LCA) of PSCs is discussed in Section 7 before arriving at the conclusions and outlook (section 8). This review aims to provide an overview of the materials and fabrication technologies which can be adapted to achieve fabrication of a greener perovskite solar cell, relevant to the current times when perovskite solar cell technology is evolving from lab to fab.

## 2. Categorization and key aspects in the quest for green solvent alternatives

### 2.1 General definitions and classification of green solvents according to Safety (S), Health (H), Environment (E)

Currently, many industries and research communities are addressing the effect of solvents on the health, safety, contamination, energy consumption, air quality and climate change.^17^ The European Chemical Agency, through REACH legislation (Registration, Evaluation, Authorization, and Restriction), targets chemical legislation to protect human health and the environment regulating substances of very high concern (SVHC) to progressively replace them with less toxic alternatives that are feasible, both technically and economically.^22^ Green solvents are environment-friendly solvents or bio solvents that make a product or process less taxing on the environment over its entire life cycle.^23^ General guidelines for the identification and selection of green solvents are based on the following factors: (i) risk factors which are analyzed using Environment, Health and Safety (EHS) methods and (ii) the energy required to produce and recover the solvent which is estimated using life cycle analysis (LCA) methods.^24,25^

According to the twelve principles of green chemistry, when considering the sustainability of a process, a solvent should possess the following features to be considered green:^24,26^

• A solvent must have negligible or reduced toxicity to minimize risks when manipulated or accidently released in nature.

• A solvent must be inert in ambient conditions (to avoid reaction/decomposition risks) and non-flammable, making it easy to store and transport.

• Synthesis of solvents must have a high atom economy percentage (processes should be designed so that the maximum amount of raw materials ends up in the product and a minimum amount of waste is produced)^24^ and must follow an energy-saving process using substances obtained from renewable feedstocks.

• A solvent must be efficiently recyclable.

• It must be highly biodegradable (degradation through biological action)^26^ without producing toxic metabolites.

Furthermore, to be used in industries, a solvent should be available on a large scale, be affordable and meet the requirements for a specific application (*e.g.*, vapour pressure, viscosity, polarity, solubilization, stabilization of the dissolved species, ability to favor a chemical process *etc.*). When all these requirements are taken into consideration, it becomes clear that no universal green solvent exists.^26^

**Fig. 2:**
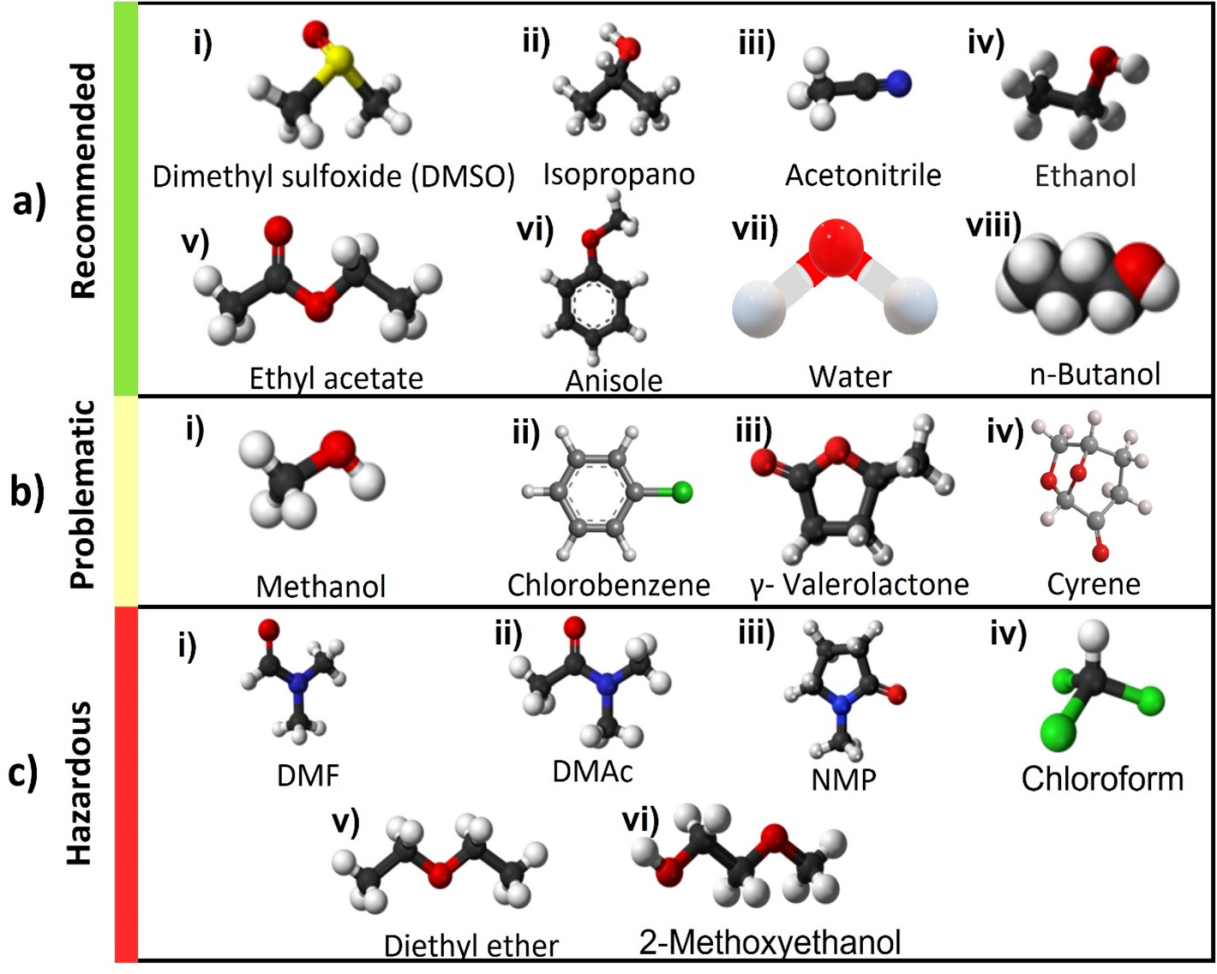
Chemical structure of various solvents used in the perovskite films classified based on outcome of Table 1 in the three categories recommended in green, problematic in yellow and hazardous in red^33^ (a) recommended solvents, (i) dimethyl sulfoxide (DMSO), (ii) IPA, (iii) acetonitrile (ACN), (iv) ethanol, (v) ethyl acetate, (vi) anisole, (vii) water, (viii) *n*-butanol, (b) problematic solvents, (i) methanol, (ii) CB, (iii) γ-valerolactone (GVL), (iv) Cyrene^39^, (c) hazardous solvents, (i) DMF, (ii) dimethylacetamide (DMAc), (iii) *N*-methyl-2-pyrrolidone (NMP), (iv) chloroform (CF), (v) diethyl ether (DE), (vi) 2-methoxyethanol (2-ME).

There have been various attempts to categorize solvents based on Safety (S), Health (H), Environment (E) scores which include Global Harmonized System (GHS) and European regulations. Several pharmaceutical companies, such as GlaxoSmithKline (GSK),^27^ Pfizer,^28^ Astra Zeneca^29^ and Sanofi,^30^ Charnwood Technical Consulting Ltd and the GCCE have ranked the sustainability of (organic) solvents and produced guides^31^ for selecting greener alternatives, in order to make evaluation and choice as simple as possible for the final user.^32–35^ Their efforts converged in 2016 with the publication of the CHEM21 (Chemical Manufacturing Methods for the 21st Century Pharmaceutical Industries) selection guide.^36^ In Table 1 and Fig. 2 we report list of solvents and their molecular structure respectively from the large CHEM21 database that are used in the fabrication of PSCs. The consensus is that simple alcohols, ketones, esters, and water should replace halogenated and polar aprotic solvents wherever possible. Conventional solvents produced from renewable sources, such as cellulose and starch, are also appropriate green candidates.^23,35^ This category comprises bio-based common solvents, such as bioethanol or bio-acetone, and recently assessed bio-renewable solvents, such as 2-methyltetrahydrofuran, γ-valerolactone (GVL) and dihydrolevoglucosenone (Cyrene).^37–39^

**Table 1:** List of solvents used to deposit ETL, perovskite, and HTL films classified based on Safety (S), Health (H), Environment (E) scores from the CHEM21 solvent guide of “classical” solvents, green for 1–3, yellow for 4–6, and red for 7–10.^33^ The higher the number the worst the score^a^

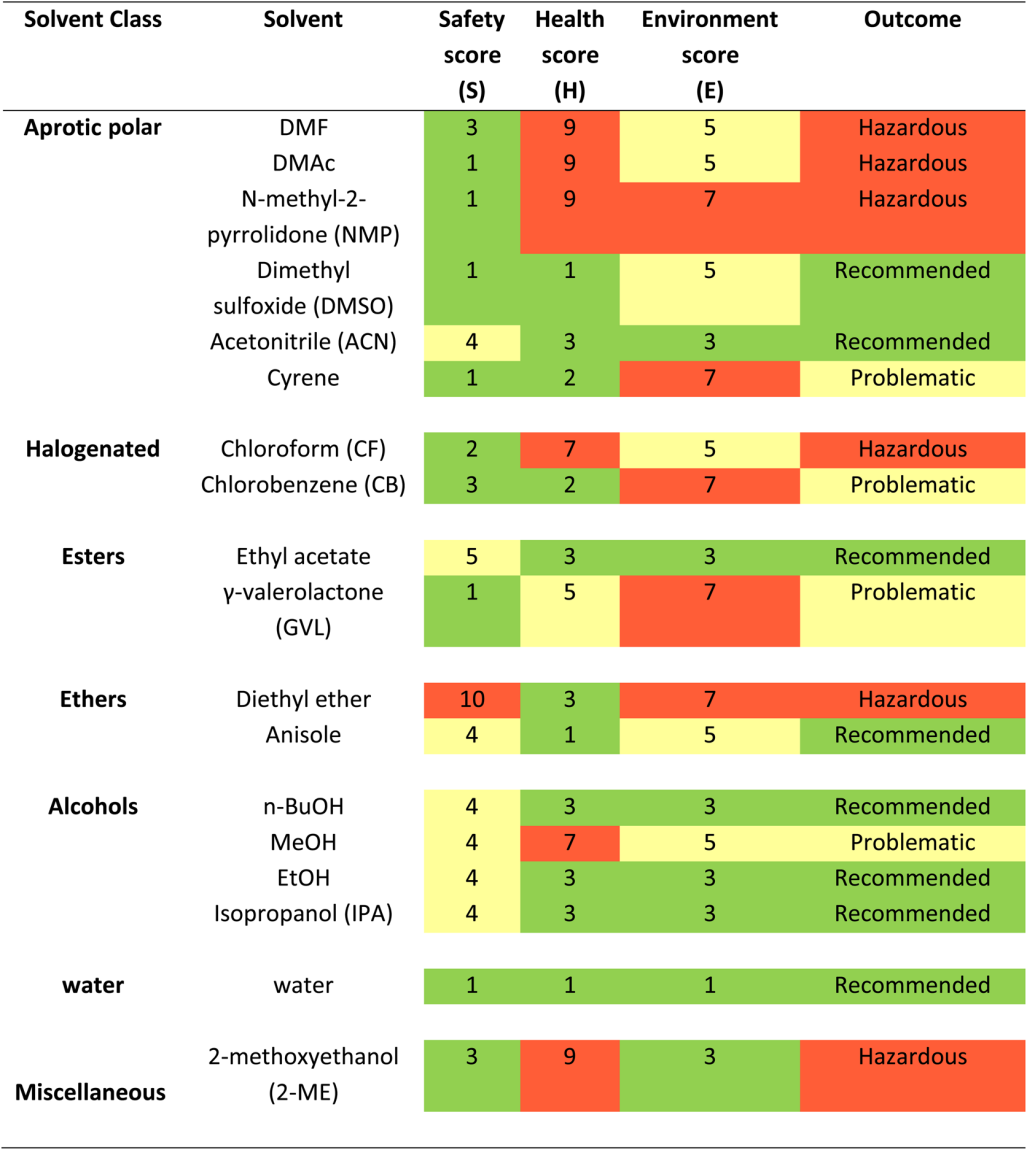

a The final ranking is based on H, S, and E scores and other parameters like H phrases or occupational exposure limits, which are not listed here.

Apart from the standard organic solvents, such as those reported in the Table 1, alternative classes of solvents called “neoteric” exist, which are considered green according to one or more favorable features including being non-volatile, and generally easily-recovered at the end of the process.^35,40^ Neoteric solvents comprise three classes of non-standard solvents: ionic liquids (ILs), deep eutectic mixtures, and supercritical fluids. Even if applications of such materials as a medium for the synthesis and processing of materials for energy have been documented,^41^ the very nature of such derivatives makes them highly impractical for the preparation of inks. ILs and deep eutectic solvents (as well as the closely associated polymeric solvents) are non-volatile.^42–44^ Supercritical fluids can only be used under relatively high-pressure conditions. The only exception regards the use of methylammonium carboxylates (such as formate, acetate, or propionate) as solvents for the preparation of the perovskite layer. In this case, however, these liquid salts do not only act as the solvent since the methylammonium cations are also incorporated in the final perovskite layer. Section 3.4 is specifically dedicated to this topic. It is however worth mentioning that ILs are gaining attention especially as additives in fabrication of PSCs, specifically to improve device stability.

### 2.2 Solubility considerations and solvent parameters useful for the selection of green solvents

For the preparation of perovskite precursor inks, as well as the transport layers, which need to dissolve in orthogonal solvents,^45^ the most critical parameter to bear in mind is the solubility in the solvent of choice. Many times, trial and error approaches are applied to find appropriate solvents (or solvent mixtures). The most common rule of thumb has always been “likes dissolves like”, meaning that solute and solvent should display similar polarity. Polarity scales exist based either on physical constants (such as the dielectric constant or the dipole moment), or on empirical constants (such as Hansen solubility parameters (HSP), the normalized polarity parameter *E*_T_^N^ or other solvatochromic parameters); the latter are generally more predictive for solvency evaluation.^46^ Knowing and understanding solvent parameters related to polarity might be a very powerful approach to predict the capability of a liquid to actually behave as a good solvent for any specific solute. This might be particularly important in the case of green solvents, as new candidates for this role are studied and proposed with increasing frequency, to avoid trial and error evaluation campaigns.

**Fig. 3:**
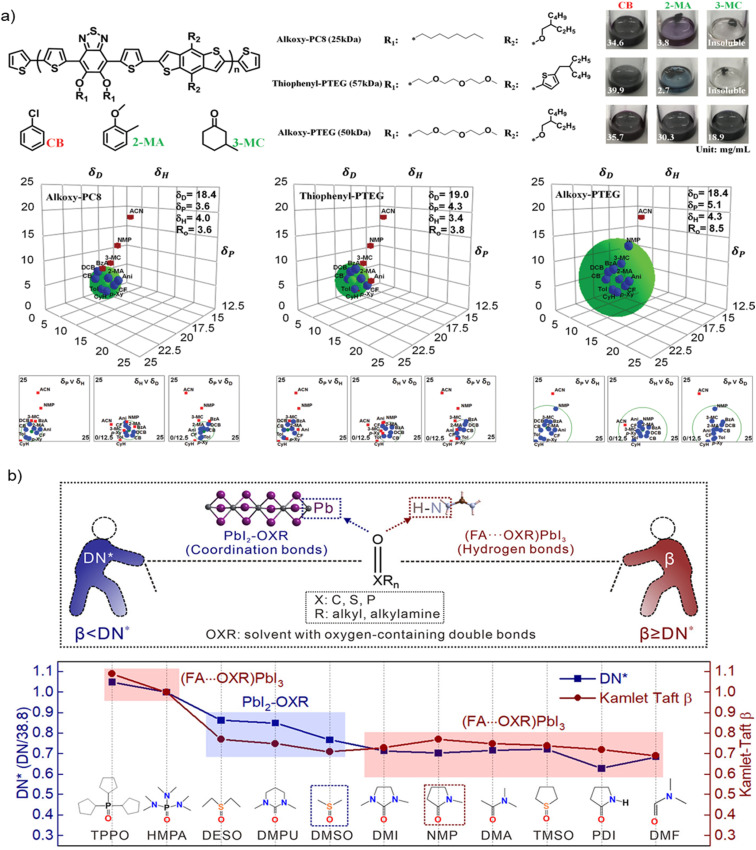
(a) Chemical structures of alkoxy-PC8, thiophenyl-PTEG, and alkoxy-PTEG; their solubility in CB, 2-methylanisole (2-MA) and 3-methylcyclohexanone (3-MC); and their HSP (and Hansen spheres), determined by dissolution in a set of different solvents, copyright 2020, WILEY-VCH Verlag GmbH & Co. KGaA, Weinheim^63^ (b) use of the normalized Gutmann's Donor Number (DN*) and Kamlet–Taft *β* parameter to rationalize the formation of PbI_2_–OXR or (FA⋯OXR)PbI_3_ structures in different solvents. When *β* < DN*, PbI_2_–OXR forms; when *β* ≥ DN*, (FA⋯OXR)PbI_3_ emerges from FAPbI_3_-based solutions, copyright 2022, American Chemical Society.^58^

Among the many possible polarity rankings based on empirical constants, HSP have already been used to rationalize the solubility of both the materials used as Hole Transport Layer (HTL) and lead-containing precursors.^17,47^Fig. 3a shows the determination of Hansen parameters *δ*_D_, *δ*_H_ and *δ*_P_ (and the Hansen sphere) for three conjugated polymers (Alkoxy-PC8, Thiophenyl-PTEG and Alkoxy-PTEG) used as HTL in FTO/SnO_2_/perovskite/HTL/Au devices (the perovskite composition being Cs_0.06_FA_0.78_MA_0.16_Pb_0.94_I_2.4_Br_0.48_). Having such information allowed the authors to deposit their HTLs using nontoxic 2-methylanisole (2-MA) and 3-methylcyclohexanone (3-MC) instead of CB. While the application of HSP to predict the solubility of neutral organic molecules and polymers is generally appropriate, in the case of perovskite precursors they display two relevant limits: they do not account for ionic interactions and solvent complexation.^48,49^ Both phenomena occur during the nucleation of the MAPbX_3_/FAPbX_3_ phase and cannot be overlooked. Indeed, it is documented that the dissolution of Pb^2+^ ions is strongly influenced by the formation of a Lewis acid–base adduct with the solvent.^50,51^

Apart from the presence of a huge dipole moment (necessary to achieve the dissolution of MAX/FAX species), a critical feature that the solvent must possess is therefore the availability of a lone pair to be donated to the Pb^2+^ ion. In short, the most suitable solvent must be a good Lewis base. Lewis's basicity can be evaluated according to different models. Both the Gutmann's donor number DN and the Mayer bond order scales have been used in the context of hybrid perovskites, with some degree of predictability.^52,53^ Unfortunately, both scales have practical limitations. The DN scale is a measure of the enthalpy of formation of a 1 : 1 SbCl_5_–base adduct in a dilute solution of an inert solvent (1,2-dichloroethane), therefore it is a solute scale and not a solvent scale. Moreover, it has been criticized for the choice of the reference Lewis acid and data reliability, reasons why other scales are now preferred, such as the Lewis affinity scale of BF_3_.^54^ Conversely, the Mayer bond order cannot be measured experimentally. Computational estimates are available, with limitations coming from a lack of experimental verification.

The solvatochromic *β*,^55,56^ a well-established parameter introduced by Kamlet and Taft to describe linear solvation energy relationships, estimates the H-bond accepting capability of a solvent. This is the equivalent of measuring the capability of the same to act as a Lewis base. *β* is a known parameter for all industrial solvents and can be measured easily for new ones, including recently introduced green alternatives.^57^ So far, the possible predictiveness of the *β* scale for the solubility of PbX_2_ and MAX/FAX species has not been directly proven, but all the solvents already used to dissolve perovskite precursors are good Lewis bases, thus substantiating the choice of this scale as a suitable molecular descriptor. In this same direction, very recently Zheng *et al.* moved a big step forward in the rationalization of solvent effects on the perovskite formation from precursors solutions.^58^ They demonstrated that, for FAPbI_3_ perovskite, the intermediate structure formed in the chosen solvent (named OXR) depend on both the capability of the solvent to coordinate with PbI_2_ (measured by DN) and to assist the formation of hydrogen bonds in (FA⋯OXR)PbI_3_ structures (measured by *β*), as shown in Fig. 3b. The disclosed solvent chemistry behind the formation of perovskite intermediate structures allowed to understand the perovskite evolution from solution and finally to prepare defect-less films and highly stable final devices.

Table 2 lists the DN and *β* values for commonly used solvents used to solubilize lead halide precursors, alongside green alternatives: triethyl phosphate (TEP), *N*,*N*′-dimethyl propylene urea (1,3-dimethyl-3,4,5,6-tetrahydro-2(1*H*)-pyrimidinone, DMPU), GVL, Cyrene and Polar Clean. TEP and DMPU are uncommon aprotic dipolar solvents of limited toxicity. GVL and Cyrene are classified as bio-renewable dipolar aprotic solvents,^59,60^ while 5-(dimethyl amino)-2-methyl-5-oxopentanoate (commercialized under the name of Rhodiasolv PolarClean)^61^ is a synthetic derivative obtained upcycling methylene glutarodinitrile (a by-product of nylon 66 manufacturing).^62^

**Table 2:** Boiling point, DN values, *β* values and OSHA hazards (Occupational Safety and Health Administration) of commonly employed solvents for lead halides dissolution (DMF, DMAc, NMP, DMSO), and recently assessed green dipolar aprotic solvents (TEP, DMPU, PolarClean, GVL, Cyrene)^a^

Solvent	Boiling point (°C)	DN (kcal mol^−1^)	*β*	OSHA hazards (as reported in safety data sheets)
DMF	153	26.6	0.69 (ref. 56)	Flammable liquids, H226
				Acute toxicity, inhalation, H332
				Acute toxicity, dermal, H312
				Eye irritation, H319 reproductive toxicity, H360D
DMAc	165–166	27.8	0.76 (ref. 56)	Acute toxicity, inhalation, H332
				Acute toxicity, dermal, H312
				Eye irritation, H319
				Reproductive toxicity, H360D
NMP	202	27.3	0.77 (ref. 56)	Skin irritation, H315
				Eye irritation, H319
				Reproductive toxicity, H360FD
				Specific target organ toxicity – single exposure, respiratory system, H335
DMSO	189	29.8 (ref. 64)	0.76 (ref. 56)	—
TEP	215	*	0.77 (ref. 56)	Acute toxicity, oral, H302
				Eye irritation, H319
DMPU	247	33.0 (ref. 58)	0.90 (ref. 65)	Acute toxicity, oral, H302
				Serious eye damage, H318
				Reproductive toxicity, H361f
Rhodiasolv PolarClean	281–282	*	0.62 (ref. 65)	Eye irritation, H319
Cyrene	227	*	0.61 (ref. 39)	Eye irritation, H319
GVL	207–208	*	0.70 (ref. 66)	—

a The asterisk * indicates that the value is unknown.

Understanding solvent–solute interactions and solvent chemistry makes possible to choose the appropriate solvent both to obtain solution of the starting materials and to find orthogonal solvents to avoid dissolution of the previously deposited layers. As such interactions are described by solvent parameters, knowing and understanding these parameters might be especially beneficial to the development of solution processed material systems and their devices. Moreover, it should be considered that the development of green solvents is a matter of intense research. The solvent parameters can therefore not only be useful to understand solvent–solute interactions and improve the quality of deposited films but might prove practical to predict the potential role of a newly developed solvents, thus facilitating the progresses in green development of PSCs.

## 3. Green solvents for the perovskite layer

Replacing toxic solvents with green solvents is one of the critical factors for commercialization of PSCs. This section discusses efforts towards greener approaches for depositing the main perovskite film. First, we briefly cover the standard solvents for lead halide perovskite precursors, *i.e.*, DMSO and DMF, that enable the formation of high-quality films.

For the preparation of perovskite precursors solutions, the proper choice of solvents becomes critical. The solvent in fact is not only a medium of the reaction, but it plays a fundamental role in the crystallization of the material, thus affecting its final morphology, defect densities, and performance when integrated in a device. So far, aprotic dipolar solvents have given the best results: DMF,^67^ NMP,^68–70^ GBL, DMSO,^71,72^ dimethylacetamide (DMAc), *etc.* They have long been popular choice due to their capability to dissolve the lead-containing precursor, relatively low volatility enabling morphological evolution on the substrate and miscibility with most other organic solvents (widening the choice for the anti-solvent).

According to research by Hamill *et al.*^53^ the DN shows a better correlation with the precursor solute's capacity to dissolve. The lead halides can be efficiently dissolved by the solvents with a DN more than 18 kcal mol^−1^. This offers a standard by which to select appropriate solvents for making the precursor solution. Aprotic polar solvents can only successfully dissolve cations because they are unable to form hydrogen bonds^73^ The strength of the aprotic solvent's bonding to Pb^2+^ and the stability of the complex are both influenced by its polarity. This will significantly affect the quality and morphology of films. Despite their outstanding capacity to dissolve the perovskite precursors, all of them pose specific sustainability issues. DMF, NMP and DMAc are known reprotoxic substances (*i.e.*, they damage human fertility) and are carcinogenic. Upon consumption, GBL is metabolized as γ-hydroxybutyric acid (GHB), a psychoactive drug: as a result, GBL has a limited legal status in many countries and strict regulations apply for its use.^74^ Furthermore, DMF, DMSO and GBL are considered hazardous solvents according to the 2012 OSHA Hazard Communication Standard (29 CFR 1910.1200).^75^ DMSO is not intrinsically toxic, but it does favour the permeation through the skin of all dissolved substances.^76^

### 3.1 Partial replacement of toxic solvent with non-toxic solvent

**Fig. 4:**
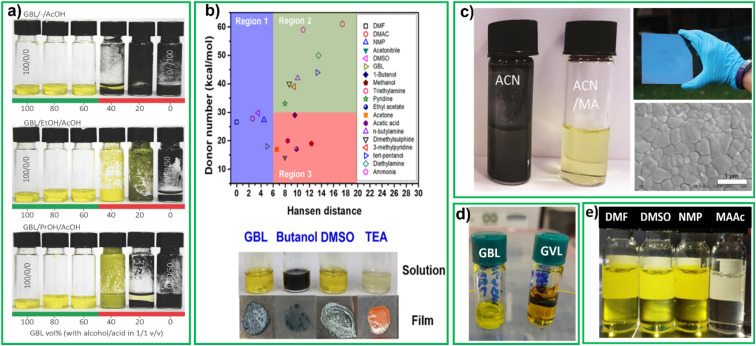
(a) Photograph of inks based on decreasing vol% of γ-butyrolactone (GBL) with equal parts alcohol/acid,^78^ reproduced with permission. Copyright 2016, Wiley-VCH, (b) plot of DN *versus* Hansen distance of different solvents related to DMF, below pictures of the perovskite precursor solutions and films prepared using different solvents reproduced with the permission^79^, Copyright 2020, American Chemical Society (c) vials of perovskite precursors CH_3_NH_3_ I : PbI_2_ (1 : 1.06 molar ratio) in neat ACN, and in the ACN/methylammonium (MA) gas mixture, perovskite coated film (above), SEM morphology of film (below), reproduced with the permission.^81^ copyright 2016, Royal Society of Chemistry, (d) GBL (left), γ-valerolactone (GVL) (right) precursor perovskite solutions, reproduced with the permission.^92^ Copyright 2021, Wiley online library, (e) perovskite solutions in DMF, DMSO, NMP and molten salt, methylammonium acetate (MAAc).^118^

In 2016 Gardner *et al.* proposed a non-hazardous compound mixture based on HSP model. The HSP can be used to identify solvents that may be compatible with perovskite precursors (CH_3_NH_3_I, PbAc_2_, and PbCl_2_) while meeting non-hazard requirements.^77^ For cyclic carbonates, like GBL, greater solvent polarity enables higher salt miscibility, which can lead to denser film. Nevertheless, these solvents have high viscosity and boiling points, causing prolonged evaporation during the annealing process. By combining high boiling point cyclic carbonates with low flash point protic solvents like EtOH and PrOH, solubility can be improved, drying temperatures can be reduced. The work of Garder *et al.* showed that it is possible to design a safe and effective solvent system by using co-solvents engineering to overcome the drawbacks of individual solvents. This study examined the all the co-solvents systems with PbAc_2_/PbCl_2_/MAI precursors. Inks are considered soluble if their precursors dissolve at a concentration of at least 1 M, and insoluble if they precipitate at lower concentrations. They concluded that inks made with GBL/alcohol/acid are soluble when they contain at least 50 vol% GBL (Fig. 4a (ref. 78)) but show colloidal separation if the GBL content is below this threshold. The ink system with GBL, alcohol, and acid mixtures for perovskite inks are considerably less toxic than conventional alternatives. The best results, with a PCE of 15.1%, were obtained using a 60/20/20 vol% mixture of GBL, ethanol, and acetic acid.

Huang *et al.* carried out a systematic study of HSP and DN of solvents used in cell fabrication.^79^ Based on solubility of perovskite precursors, solvents can be divided into the three regions shown in Fig. 4b. Solvents located in region 1 exhibit a low Hansen distance Ra (compared to DMF) and they can dissolve perovskite materials well, regardless of their DN values. With increasing Hansen distance, the effect of the DN values on the solubility of perovskite materials became obvious gradually. The solvents located in region 2 showed acceptable solubility for perovskite precursors with a DN value larger than 30. Finally, solvents in region 3 (with high Ra (>6) and low DN (<30)), showed poor solubility for perovskite precursors. The results indicate that CH_3_NH_3_I and PbI_2_ have good solubility in GBL, DMSO and TEA, as the solutes formed a clear solution in these solvents (see lower Fig. 4b). The high Ra (9.60) of 1-butanol indicates that it is a poor solvent for PbI_2_ resulting in a dark opaque solution. The authors determined that the solvent mixture that gave the highest efficiency was DMSO : GBL in 9 : 1 by volume. This mixture contains 90% of DMSO that is considered non-toxic according to the CHEM21 of 2016 solvent selection guide. Cells fabricated by blade coating in air with this mixture together with a green additive called polyethylene glycol (PEG) which passivated defects in perovskite film delivered a PCE of 17.0%.^79^

Replacement of solvents for perovskite precursor with, at least in part, non-toxic alternatives, leads to more difficulties in reaching high quality crystallization. Researchers have thus introduced gases, together with nontoxic solvents to improve film quality, even without further antisolvent treatment. For the first time Cui *et al.* reported methylamine induced defect-healing (MIDH) in CH_3_NH_3_PbI_3_ perovskite thin films. This process involves the ultrafast and reversible reaction of the films with CH_3_NH_2_ gas at room temperature, leading to the formation of defect-free films. This study provided a new approach in the development of hybrid perovskite films.^80^ Noel *et al.* study demonstrated the use of low boiling, low viscosity ACN/methylamine gas (MA) composite solvent system for dissolving CH_3_NH_3_PbI_3_. Upon spin-coating this precursor solution, the researchers obtained extremely smooth and pinhole-free perovskite films that crystallize at room temperature. The solution contained a 1 : 1.06 ratio of MAI and PbI_2_ at a concentration of 0.5 M. After adding ACN, a black dispersion was observed (left vial, Fig. 4C). Subsequently, MA gas was bubbled through such dispersion until all the perovskite particles dissolved and formed a clear, light-yellow solution (right vial, Fig. 4C). The solution was spin coated onto a substrate resulting in uniform dense, smooth mirror-like film (top right, Fig. 4C). The SEM image (bottom right, Fig. 4C) shows individual domains that range from 500 nm to 700 nm in size. This study represented the first attempt to use a low-boiling point solvent for fabrication of large-area perovskite films with the one-step method.^81^ Other studies have shown enhanced carrier lifetime, crystallinity, and grain size through use of MA and ethylamine (EA) gas assisted recrystallization of methylammonium lead iodide films.^16,82,83^

Here we mentioned some more studies involved mixed non-volatile, coordinating solvents (NVCS) like DMF, DMSO and volatile, non-coordinating solvents (VNCS) like ACN, 2-ME, GBL *etc.* Normally coordinating solvents have strong bonding between perovskite precursor materials, particularly Pb^2+^. This strong bonding between the solvents and Pb^2+^ through the use of NVCS leads to the formation of a solid-state intermediate phase.^84^ Deng *et al.* studied the trade-off between rapid crystallization and large grain growth through solvent selection. Inks developed with combinations of VNCS (2-methoxy ethanol (2-ME) and ACN) and NVCS solvents like DMSO were used to fabricate modules *via* blade coating delivering a PCE of 16%.^84^ Further, Li *et al.* suggested that a 7 : 3 volume/volume composition of DMSO and ACN works best.^85^ Numata *et al.* fabricated formamidinium lead-bromide (FAPbBr_3_) perovskite absorber film made using a sequential deposition technique using a DMSO and tetramethylenesulfoxide (TMSO) mixed solvent, PSCs with an open-circuit voltage (*V*_OC_) of over 1.5 V were developed.^91^ Guerrero *et al.* optimized GBL and DMSO mixtures to enable blade-coating deposition.^86^ Galagan and co-workers demonstrated that a combination of DMSO, 2-methyl pyrazine and 1-pentanol was safer for industrial scale-up than devices showing comparable performances to ones processed in DMF.^87^ In 2020, Zhang *et al.* presented a double-cation perovskite solution that was deposited using pure dimethyl sulfoxide (DMSO) and fabricated 21.8% certified efficiency device.^88^ In 2022, X. Cao *et al.* introduced a green solvent called triethyl phosphate (TEP) to prepare perovskite solutions, and the non-toxic solvent dibutyl ether (DBE) as the anti-solvent and developed ((FAPbI_3_)_1−*x*_(MAPbBr_3_)_*x*_) based perovskite cells with a PCE of 20.1%.^89^

HSP and DN can help find suitable solvent or co-solvent systems as (partial) replacements for toxic solvents. By combining the right amount of high boiling point cyclic carbonates such as GBL with low flash point protic solvents like ethanol or propanol, the solubility is enhanced and the time for solvent evaporation can be reduced.^17^ Mixing low boiling point, low viscosity solvents with highly evaporative gases like MA gas or EA gas can lead to quick and room temperature crystallization of perovskite films. Alkylamines (EA or FA) can dissolve perovskite salts when in used in tandem with aprotic solvents like ACN.^81^ Using volatile solvents such as ethanol, tetrahydrofuran, or ACN often results in uncontrolled nucleation and formation, limiting the grain growth of perovskite. Therefore, a careful optimization of the solvent–antisolvent system is necessary for good perovskite film formation. We have discussed suitable anti solvent for high quality of perovskite film formation in Section 3.5. Mixture of non-volatile coordinating solvents (DMSO) and volatile non-coordinating solvents (ACN, GBL, 2-ME) helps rapid crystallization of perovskite films, which is useful for scaling up perovskite technology. Solvents systems like DMSO/ACN, DMSO/2-methyl pyrazine/1-pentanol and DMSO/TMSO and triethyl phosphate (TEP) are viewed as safer options compared to conventional solvents used for ink formulation. In 2020, Zhang *et al.* presented a double-cation perovskite solution that was deposited using pure dimethyl sulfoxide (DMSO) and fabricated a solar cell with a certified efficiency of 21.8%.^88^ But this study still used toxic antisolvents like CB or DEE which need to be replaced. Following Section 3.2 will describe ways in which research has processed perovskite films with only one solvent (and without antisolvent). Table 3 provides a concise overview of absorber materials, solvent choices, deposition techniques, device architectures, and key photovoltaic parameters for perovskite solar cells (PSCs) using environmentally friendly or low-hazard solvents in their fabrication.

**Table 3:** Absorber material, solvents, deposition method, device architecture, and photovoltaic parameters of reported perosvkite solar cells fabricated with non-hazardous or less-hazardous solvents for the perovskite layer

Absorber	Deposition method	Solvent	Device architecture	*V*_OC_ [V]	*J*_SC_ [mA cm^−2^]	FF [%]	PCE [%]	Ref.
**Solvent mixtures**								
MAPbICl_2_	Spin-coating	GBL/ethanol/acetic acid (60/20/20 vol%)	Glass/ITO/TiO_2_/perovskite/spiro-OMeTAD/Au	0.88	21.2	71	15.1	17
MAPbI_3_	Doctor blade coating	3 : 2 (GBL : DMSO)	Glass/FTO/TiO_2_/perovskite/spiro-OMeTAD/Au	1.01	20.6	75	15.6	86
CH_3_NH_3_PbI_3−*x*_Cl_*x*_	Spin-coating	DMSO/2-MP/1-P	Glass/ITO/C–TiO_2_/perovskite/spiro-MeOTAD/Au	1.04	22	72	16.5	87
MAPbI_3_	Spin-coating	ACN + MA gas	FTO/TiO_2_/C60/perovskite/spiro-OMeTAD/Au	1.1	22.1	77	19	81
MAPbI_3_	Blade coating	ACN (60%, v/v)/2-ME (40%, v/v)	Glass/ITO/PTAA/perovskite/C_60_/BCP	1.13	23	81.8	21.3	84
CH3NH_3_PbI_3−*x*_Cl_*x*_	Spin-coating	DMSO + ACN (7 : 3)	Glass/FTO/C–TiO_2_/m-TiO_2_/perovskite/spiro-MeOTAD/Au	1.03	20.39	73	15.32	85
MAPbI_3_	Spin-coating	DMF + ACN	Glass/ITO/C–TiO_2_/perovskite/spiro-MeOTAD/Au	1.11	22.49	75.09	18.8	90
MAPbI_3_	Blade coating	DMSO + GBL (9 : 1 v/v)	Glass/FTO/NiO_*x*_/perovskite/PCBM/C60/BCP/Ag	1.09	21.29	73.49	17.02	79
FAPbBr_3_	Two step	DMSO + TMSO	FTO/TiO_2_/Li–m-TiO_2_/FAPbBr_3_/PMMA/spiro/Au	1.53	6.96	74.0	7.88	91
FA_1−*x*_MA_*x*_PbI_3_	Spin-coating	DMSO	FTO/SnO_2_/m-TiO_2_/perovskite/spiro-OMeTAD/AU	1.09	22.39	79.5	21.76	88

**Single solvents**								
(FAPbI_3_)_1−*x*_(MAPbBr_3_)_*x*_	Spin-coating	Triethyl phosphate (TEP)	FTO/SnO_2_/perovskite/spiro-OMeTAD/Au	1.09	20.13	74.8	20.13	89
MAPbI_3_	Blade coating	GVL	Glass/FTO/C–TiO_2_/m-TiO_2_/perovskite/carbon	0.89	23.42	62	12.91	92
FA_0.83_Cs_0.17Pb_(I_0.87_Br_0.13_)_3_	Blade coating	DMSO	Glass/ITO/PTAA/SiO_2_ NPs/perovskite/PCBM/BCP/Ag	1.10	19.6	76.9	16.7	93
CsPbIBr_2_	Spin coating	DMSO	FTO/c-TiO_2_/perovskite/spiro-OMeTAD/Ag	1.22	13.33	71.0	11.49	94
FA_0.85_MA_0.15_PbI_2.55_Br_0.45_	Blade coating	DMSO	FTO/C–TiO_2_/perovskite/PTAA/Ag	1.10	23.20	78.58	20.05	95
FA_0.85_MA_0.15_PbI_2.55_Br_0.45_	Blade coating	DMSO	ITO/PTAA/perovskite/PCBM/BCP/In	1.10	22.34	78.26	19.23	95
FA_0.75_Cs_0.25_PbI_2.7_Br_0.3_	Blade coating	DMSO	FTO/SnO_2_/perovskite/spiro/Au	1.14	23.4	75.6	20.2	96

**H**_**2**_ **O as co-solvent**								
MAPbI_3_	Spin-coating/IPA bath	Pb(NO_3_)_2_·H_2_O & MAI·IPA	Glass/FTO/C–TiO_2_/m-TiO_2_/perovskite/spiro-MeOTAD/Au	0.94	21.81	61	12.58	97
MAPbI_3_	Spin-coating/IPA bath	Pb(NO_3_)_2_·H_2_O & MAI·IPA	Glass/FTO/C–TiO_2_/m-TiO_2_/perovskite/spiro-MeOTAD/Au	1.07	19.07	74	15.11	98
MAPbI_3_	Spin-coating/IPA bath	Pb(NO_3_)_2_·H_2_O & MAI·IPA	Glass/FTO/C–TiO_2_/m-TiO_2_/perovskite/spiro-MeOTAD/Au	0.9	21.2	72	13.7	99
Cs_0.1_FA_0.9_Pb(I_0.83_Br_0.17_)_3_	Spin-coating/IPA bath	H_2_O & IPA	Glass/FTO/TiO_2_/perovskite/spiro-MeOTAD/Au	0.96	18.8	66	11.7	100
FA_*x*_MA_1−*x*_I_0.9_Br_0.1_	Spin-coating/IPA bath	H_2_O &IPA	PEN/SnO_2_/TiO_2_/perovskite/spiro-MeOTAD/Au	1.03	21.77	73.6	16.5	101
MAPbI_3_	Spin-coating/IPA bath	H_2_O & IPA	Glass/FTO/C–TiO_2_/m-TiO_2_/perovskite/spiro-MeOTAD/Au	1.1	22.5	74	18.3	102
FA_*x*_MA_1−*x*_I_0.9_Br_0.1_	Chemical bath & spin coating	H_2_O & IPA	Glass/FTO/C–TiO_2_/m-TiO_2_/perovskite/spiro-MeOTAD/Au	0.918	21.61	56.57	11.23	103

**Ionic liquids (ILs)**								
α-FAPbI_3_	Spin coating	MAF	ITO/SnO_2_/perovskite/spiro-OMeTAD/MoO_3_/Au	1.17	25.34	81.36	24.1	104
(MA_0.15_FA_0.85_)Pb(I_0.85_Br_0.15_)_3_	Spin coating	MAP/DMSO/ACN	Glass/FTO/C–TiO_2_/m-TiO_2_/perovskite/spiro-MeOTAD/Au	1.07	23.08	62	15.46	105
MAPbI_3_	Spin coating	MAP	ITO/SnO_2_/perovskite/spiro-OMeTAD/MoO_3_/Ag	1.12	23.39	79.52	20.56	106
MAPbI_3_	Spin coating	MAAc	ITO/SnO_2_/perovskite/spiro-OMeTAD/MoO_3_/Ag	1.07	23.12	76.65	18.99	106

**Anti-solvents**								
MAPbI_3_	Spin coating	Tetraethyl orthocarbonate (TEOC)	Glass/ITO/SnO_2_/perovskite/spiro-OMeTAD/PCBM/BCP/Ag	1.06	21.9	78	18.15	107
[CsPbI_3_]_0.05_[(FAPbI_3_)_0.85_(MAPbBr_3_)_0.15_]_0.9_	Spin-coating	Anisole	Glass/FTO/TiO_2_/perovskite/spiro-OMeTAD/Ag	1.15	21.98	78	19.76	108
(FAPbI_3_)_1−*x*_(MAPbBr_3_)_*x*_	Spin coating	Anisole	PET/ITO/SnO_2_/perovskite/spiro-OMeTAD/Au	1.11	22.48	68.49	17.09	109
Cs_0.05_(MA_0.17_FA_0.83_)_0.95_Pb(I_0.83_Br_0.17_)_3_	Spin-coating	Anisole	Glass/FTO/TiO_2_/perovskite/spiro-OMeTAD/Au	1.14	23.5	76	20.14	110
(FA_0.85_MA_0.15_)Pb(I_0.85_Br_0.15_)_3_	Spin coating	Ethyl acetate	Glass/FTO/SnO_2_/perovskite/spiro-OMeTAD/Au	1.22	23.13	73.7	20.77	111
(FA_0.85_MA_0.15_)Pb(I_0.85_Br_0.15_)_3_	Spin coating	Methyl benzoate (MB)	Glass/FTO/SnO_2_/perovskite/spiro-OMeTAD/Au	1.21	24.46	74.95	22.37	111
(FA_0.85_MA_0.15_)Pb(I_0.85_Br_0.15_)_3_	Spin coating	Ethyl acetate	Glass/FTO/C–TiO_2_/m-TiO_2_/perovskite/spiro/Au	1.12	22.89	75.6	19.43	112

### 3.2 Non-toxic solvents which can be used pure

Till now we have discussed co-solvents used to dissolve perovskite precursors the following paragraphs are going to describe various single solvents which can dissolve perovskite precursors by itself without any co-dependency. In the life cycle assessment study shown by Vidal *et al.*^113^ DMSO had the least human health and environmental impact compared to DMF, DMAC, NMP, DMEU, GBL, THF, and DMPU. DMSO is able to solubilize perovskite precursors due to its high DN (29.8 kcal mol^−1^). From CHEM21 Table 1 and Vidal LCA studies, DMSO can be considered a green solvent for perovskite device fabrication even though we noted above that it assists permeation through the skin of dissolved substances. Küffner *et al.*^93^ reported blade-coated devices in a single step process for inverted PSCs (p–i–n). The deposition process utilized only environmentally friendly green DMSO and carried out at low temperatures, which is making it a significant advancement towards commercialization of solution processed of PSCs. To produce the perovskite dry film from a wet film a state of supersaturation must be induced in the film. This is achieved through the rapid drying or quenching of the film. To do this there four widely used quenching methods are antisolvent, vacuum, heat, and gas-assisted quenching.^114^ Recently many of researchers reported highly efficient perovskite films by blade-coating followed by gas quenching.^84,93,115^ Jafarzadeh *et al.* used a two-step blade coating technique to deposit perovskite using DMSO as the solvent for the first step.^116^ They demonstrated that gas quenching in combination with additive engineering enables the deposition of perovskite on flexible substrates under ambient conditions without the usage of high deposition temperature. The all-blade coated flexible perovskite solar cells delivered 14% PCE.

Xing *et al.* reported (FA)–cesium lead halide perovskite by heat-assisted blade coating with DMSO as a solvent. The use of DMSO has been shown to enhance the formation of α-phase crystals, resulting in improved crystallinity. Additionally, increasing the substrate temperature during the coating process leads to a more compact film, with a more pronounced preferred facet orientation and a desired phase transition.^96^ He *et al.* reported a new method in blade coating of perovskite films called meniscus-assisted solution printing (MASP) and showed dense perovskite films with large grains with pure DMSO as solvent. In this study they achieved a PCE of 19.23% in inverted planar and PCE of 20.05% in standard planar perovskite devices.^95^

In 2021, Worsley *et al.* introduced a new biodegradable green solvent, GVL, often used in foods and perfumes, which was as an alternative to GBL. PSCs fabricated with GVL delivered a PCE of 12.9% on a 1 cm^2^ active area (GBL and GVL perovskite solutions shown in Fig. 4d).^92^ According to CHEM21 solvent selection guide GVL is considered as problematic solvent. Many bio-based solvents are considered problematic because they have a high boiling point, making it challenging to separate the product and recycle the solvent. Some new solvents, such as γ-valerolactone, are only produced in limited quantities or only as intermediates, and have not yet been evaluated by REACH. As a result, these solvents are given a default score of at least 5 in health and environmental criteria.^33^ Recently, Kerkel *et al.* extensively studied HSP and COSMO-RS calculated on GVL. Toxicity tests on aquatic plants, bacteria, invertebrates, and a vertebrate cell line showed that GVL has low acute toxicity to aquatic organisms. Furthermore, GVL was found to be readily biodegradable, further reinforcing its potential as a environmentally friendly solvent.^117^ Some common perovskite solutions with DMF, DMSO, NMP and molten salt (MAAc) shown in Fig. 4e.^118^

### 3.3 Water (H_2_O) in manufacturing of PSCs

#### 3.3.1 Improved PSCs performance adding water (H_2_O) in the precursor solution

**Fig. 5:**
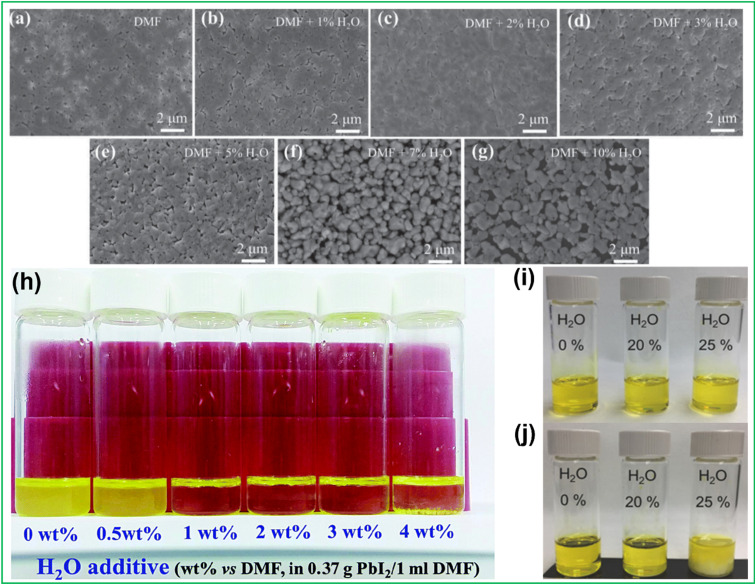
SEM images of CH_3_NH_3_PbI_3−*x*_Cl_*x*_ films based on (a) DMF, (b) DMF + 1% H_2_O, (c) DMF + 2% H_2_O, (d) DMF + 3% H_2_O, (e) DMF + 5% H_2_O, (f) DMF + 7% H_2_O, and (g) DMF + 10% H_2_O solvent.^119^ copyright 2015, Wiley online library (h) the photographs of PbI_2_/DMF solutions containing various amounts of H_2_O additive (the H_2_O content is volume ratio *vs.* DMF)^67^, copyright 2015, The Royal Society of Chemistry, (i) & (j) photograph of perovskite (CH_3_NH_3_PbI_3−*x*_Cl_*x*_) precursor solutions of fresh and overnight stored perovskite solutions with various H_2_O concentrations, reproduced and copyright 2018,^120^ published by Wiley-VCH.

Studies mentioned in the previous section reported successful fabrication of PSCs using less toxic solvents such as ACN, alcohols, MA gas and 2-ME. Nevertheless, these solvents are low boiling point solvents, potentially explosive bringing questions marks on their safety. Despite humidity being the worst enemy of formed perovskite films, causing their rapid degradation, water and/or humidity can improve crystallization of perovskite films and increase device performance. Gong *et al.*^119^ studied that the perovskite morphology can be effectively improved by adding 2% water, which improves surface coverage Fig. 5a–g, crystallization, and stability. Device PCE boosts from 12.13% to 16.06% and increases cell stability in ambient environments. In 2015, Wu *et al.* reported that scarce amounts of H_2_O (2 wt%) in DMF added to the perovskite precursor inks resulted in devices with a PCE of 18% (two-step perovskite film deposition), PbI_2_ has low solubility in dried dimethylformamide (DMF), resulting in a colloid suspension instead of a homogeneous solution when 0.37 g of PbI_2_ is dissolved in 1 ml of 0.80 M DMF and heated at 80 °C for 10 min. However, when a small amount of water (0.5–4 vol% *vs.* DMF) is added to the PbI_2_/DMF solution, it becomes homogeneous shown Fig. 5h. This is likely due to the fact that water is miscible with DMF and changes the polarity, dielectric constant, and solubility parameter of DMF, which brings it closer to that of PbI_2_, allowing it to dissolve fully in the mixed solvent.^67^ Furthermore, it was observed that increasing water content beyond 3 wt% (with respect to DMF) resulted in a drastic reduction of *J*_sc_. Liu *et al.* demonstrated one-step deposition of perovskite solutions Fig. 5i and j photographs depict homogeneous and clear solutions of perovskite precursor with varying water content in the total solvent system. However, when storing the precursor overnight, the 25% water content precursor (H_2_O-25% precursor) forms precipitate, which can be redissolved by heating. To prevent continuous clustering, the 20% water content precursor (H_2_O-20% precursor) was chosen as the primary point of comparison in the study, while higher concentrations (H_2_O-25% precursor) were only used for comparison without storage. Presence of water in perovskite solution can actually enable humidity tolerance during fabrication in air and solar cells with power conversion efficiency of 20% were obtained.^120^ Although the freshly made solution with 25% H_2_O appeared clear initially, the solution turned turbid after ageing for about 8 hours. This study concluded that water content in perovskite solution cannot be increased beyond 25% (by volume with respect to DMF).

#### 3.3.2 Perovskite precursors with water as a solvent

“If the plan doesn't work, change the strategy”: rather than attempt to dissolve known precursors in green solvents, researchers have developed new precursors that are soluble. Lead nitrate [Pb (NO_3_)_2_] has been shown to easily dissolve in water. The other precursors that are required to prepare perovskite inks, such MAI, can easily dissolve in IPA or other non-toxic solvents.

**Fig. 6:**
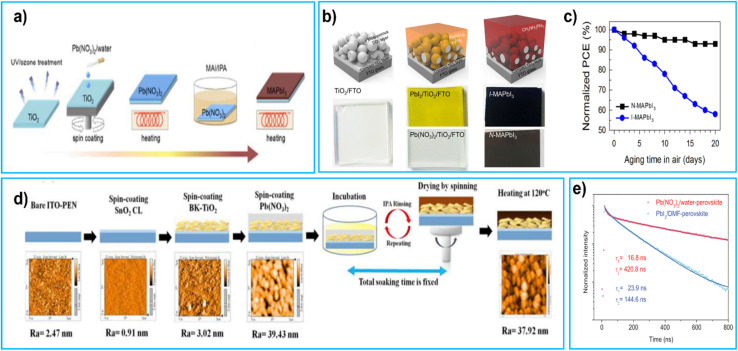
(a) Procedure for preparing MAPbI_3_ layer in the Pb(NO_3_)_2_/water system, reproduced with permission, Copyright 2015, The Royal Society of Chemistry,^97^ (b) schematic representation conversion of MAPbI_3_ formation from aqueous lead nitrate, structural schemes and optical views of mesoporous-TiO_2_, Pb (NO_3_)_2_ and PbI_2_, and MAPbI_3_ layers sequentially stacked on an FTO coated glass substrate, reproduced with permission, copyright 2017, American Chemical Society^99^ (c) normalized PCE values of the corresponding solar cells before and after storage in a dark chamber with 20% RH for various lengths of time, reproduced with permission, Copyright 2017, American Chemical Society^99^ (d) process flowchart of fabricating PEN-PSC in this study and the AFM topographic image of each step in the process, reproduced with permission, Copyright 2019, Elsevier^101^ (e) TRPL decay of the perovskite film fabricated using the Pb(NO_3_)_2_/water (red) and PbI_2_/DMF (blue) systems with biexponential decay fitting, reproduced with permission, Copyright 2020, Wiley.^102^

In 2015, Hsieh *et al.* introduced a low toxicity lead nitrate precursor [Pb (NO_3_)_2_], which can dissolve in water. By a two-step process for the deposition of the perovskite layer (shown in Fig. 6a) they achieved a PCE of 12.6%.^97^ The conversion mechanism of Pb (NO_3_)_2_ to MAPbI_3_ was considerably slower (700 s) than those with conventional precursors (20 s forPbI_2_ and MAI) needing an intermediate ion–exchange reaction.^97^[Pb (NO_3_)_2_] + 2CH_3_NH_3_I → PbI_2_ +2CH_3_NH_3_ (NO_3_)PbI_2_ + CH_3_NH_3_I → CH_3_NH_3_PbI_3_

The slow incubation process led to a dissolution–recrystallisation effect (also known as Ostwald ripening)^121^ resulting in rough and non-uniform perovskite films^98^ which increases charge carrier recombination time. To solve this problem and shorten the synthesis time (500 s), in 2018 the same group introduced chloride ions in MAI/IPA solution. These chloride ions greatly improved efficiency to 15.1% as well as stability over 55 days.^98^ In 2017, V. Shinde *et al.* reported perovskite films formed by aqueous lead nitrate ([Pb (NO_3_)_2_]/H_2_O) and perovskite conversion schematic diagram shown in Fig. 6b. The structural layout as well as visual appearance of mp-TiO_2_, layers of Pb(NO_3_)_2_ and PbI_2_, and a stack of MAPbI3 layers on FTO shown in Fig. 6b. The PbI_2_ and Pb (NO_3_)_2_ layers, when coated on TiO_2_ surfaces through spin-coating, appear yellow and colourless respectively. The perovskite films are formed by combining different lead sources with MAI dissolved in IPA. The process of converting Pb(NO_3_)_2_ to N-MAPbI_3_ (with Pb(NO_3_)_2_)is slower than the typical direct method of converting PbI_2_ to I-MAPbI_3_ (with PbI_2_)because Pb(NO_3_)_2_ undergoes an intermediate ion–exchange reaction before becoming N-MAPbI_3_ and they observed that the conversion of Pb(NO_3_)_2_ to N-MAPbI_3_ takes 600 s (10 min) while PbI_2_-based I-MAPbI_3_ system takes only 60 s. N-MAPbI_3_ based devices maintained 95% of their initial PCE over 20 days.^99^ As shown in Fig. 6c solar cells before and after storage in a dark chamber with 20% RH for various lengths of time and solar cells operated under 1 Sun illumination. In 2017, K. Sveinbjörnsson *et al.* introduced mixed cation and halide Cs_0.1_FA_0.9_Pb(I_0.83_Br_0.17_)_3_ perovskites using aqueous CsNO_3_ and Pb(NO_3_)_2_ achieving a PCE of 13.0%.^100^ In 2019, P. Zhai *et al.* used a two-step process in which, after drying Pb(NO_3_)_2_, the film was immersed in IPA solutions containing either MAI or FAI/MAI/MABr (fabrication steps shown in Fig. 6d). With this method FA_*x*_MA_1−*x*_I_0.9_Br_0.1_ PSCs with PCE = 16.50% were obtained.^101^ In 2020, T. Hsieh *et al.* demonstrated Pb(NO_3_)_2_/water-based PSCs with a PCEs of 18.3% and a large *V*_OC_ of 1.1 V, with longer lifetime compared to the cell fabricated with the PbI_2_/DMF precursor from the photoluminescence (PL) decay results showed that the Pb(NO_3_)_2_/water-based perovskite film had a fast component with a short lifetime, which represents the process of photogenerated carriers filling into subgap traps.^102^ It also had a slow component with a much longer lifetime, resulting from band-to-band recombination. The fast component corresponds to the first decay, while the slow component corresponds to the second decay as shown in Fig. 6e. In 2021, S. Gozalzadeh *et al.* used lead sulphide (PbS) produced by aqueous chemical bath deposition. Subsequently PbS films were chemically converted to PbI_2_ and finally transformed into mixed-cation mixed halide pinhole-free uniform perovskite films. The resulting cells delivered a PCE of 11.35%.^103^ Since toxic solvents have been completely substituted with eco-friendly water and IPA the results of these works are very encouraging. However, the devices processed with unconventional precursor like Pb(NO_3_)_2_ or PbS in water based solution are underperforming in comparison to conventionally processed devices. This could be possibly due to the formation of intermediate products which slows down the reaction and relative merits of the residual water during solvent evaporation and crystallization of the precursor into perovskite phase, leading to degradation of the perovskite itself.

Despite the promising results of water-based perovskite solar cells (PSCs) with a reported highest efficiency of 18.3%, reproducibility remains a major challenge in the field. Trace amounts of nitrate and residual water can lead to an increased number of defects in films. The conversion process for creating a complete perovskite film also requires a longer time frame and multiple rigorous IPA rinsing steps.

However, the use of water as a solvent in PSCs offers numerous benefits in the field of solar energy. This relatively simple method of fabrication would have several advantages compared to traditional methods, a water-based solvent that is more environmentally friendly than other solvents, as well as cost-effective.

### 3.4 Ionic liquids as solvents

ILs have been researched as a potential solvent for perovskite solar cells. ILs are liquid salts that are composed of positively and negatively charged ions. ILs refer to salts that exist in a liquid state and possess a low melting point, typically less than 100 °C.^122^ They are often used as solvents in PSCs due to their interesting chemical and physical properties, such as high conductivity, thermal stability, low vapour pressure, adjustable acidity, and high solubilizing power compared to traditional solvents.^123–126^ In addition, the role of IL in homogenous nucleation is significant as it modifies surface defects, adjusts energy levels, and influences the kinetics of crystal growth and charge transportation of the emerging PSCs fields.^127^

MAF is an effective solvent for dissolving PbI_2_ and MAI due to its ionic nature and strong solvation interactions. The COO– groups in MAF interact strongly with Pb^2+^ ions, and CH_3_NH_3_^+^ and I^−^ ions, making MAF more effective in dissolving PbI_2_ and MAI than other common organic solvents like DMF and DMSO.^128^ In 2015, Moore *et al.* reported, the first use of ILs as precursor solvents, *i.e.* methylamine formate (MAF) to deposit highly crystalline and uniform MAPbI_3_ films^129^ ILs like MAF are non-volatile because they have very low vapor pressures, meaning they do not easily evaporate at ambient temperatures. This is due to the fact that they are composed of ions rather than molecules, and the forces holding the ions together are much stronger than the forces holding molecules together in a traditional liquid. To remove MAF residue from the perovskite films they have treated perovskite films with butanol resulting highly crystallized MAPbI_3_ without any residual precursors. Cho *et al.* reported the thermal gradient-assisted directional crystallization process is a technique used for perovskite films.^128^ In this process, a solution of MAI and PbI_2_ (1 : 1 molar ratio) in MAF (25 wt%) is prepared and then spread on a substrate using blade-coating method. The liquid film is then exposed to a thermal gradient, causing the perovskite crystals to form in a specific direction. This process improves the uniformity and quality of the perovskite film.^128^

Protic Ionic Liquids (PILs) are a class of ILs that contain protons (H^+^) as part of their cation, which can participate in hydrogen bonding and other types of interactions.^130,131^ PILs possess lower thermal stability and chemical stability as compared to other classes of ILs. They also exhibit non-negligible vapor pressure, which can be considered an advantage when it comes to solution processing of hybrid perovskites.^132^ This is because the removal of residual solvents is a critical criteria for achieving high material purity in perovskite solar cells. The vapor pressure of PILs allows for easy removal of the solvent by simple evaporation, which reduces the risk of contamination and impurities in the perovskite film.^105^ Additionally, PILs can also be chosen based on their solubility and ability to dissolve the precursors of perovskite, which can help to improve the quality and uniformity of the perovskite film. It's worth mentioning that the choice of IL and its properties depend on the specific application and the perovskite material used, and it is important to carefully consider the trade-offs between the properties of the IL and the requirements of the application. PILs have a high viscosity that can make it difficult to create uniform thin films using spin-coating at ambient temperatures. One way to overcome this challenge is to blend the PIL with a co-solvent, such as water, ethanol, isopropanol, or ACN. These co-solvents can help to lower the viscosity of the ink, making it easier to process into a uniform thin film.

**Fig. 7:**
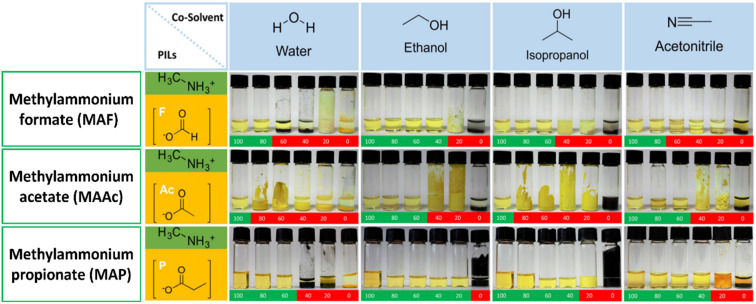
Photographs of binary perovskite ink-systems (protic ILs/co-solvent) with increasing co-solvent volume. (Top-row, methylamine formate (MAF), middle-row, methylammonium acetate (MAAc), bottom-row, methylammonium propionate (MAP).) Green indicates: soluble, red indicates: separation or formation of solids^105^, reproduced, copyright 2018 published by Elsevier Ltd.

**Fig. 8:**
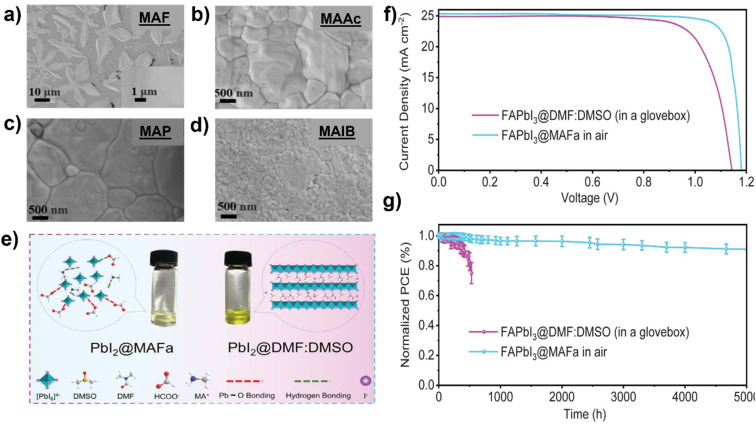
SEM images of the perovskite films fabricated by (a) MAF, (b) MAAc, (c) MAP, and (d) MAIB solvents,^106^ reproduced and copyright 2022, American Chemical Society, (e) images of PbI_2_-MAF and PbI_2_-DMF : DMSO solutions and schematic diagram of interactions in the solutions^104^ reproduced and copyright 2021, Science. (f) *J*–*V* curves of champion device based on FAPbI_3_@MAF and FAPbI_3_@DMF : DMSO films, (g) stability of unencapsulated devices stored in an N_2_-filled glove box in the dark,^104^ reproduced and copyright 2021, Science.

S. Öz *et al.*^105^ prepared perovskite solutions using Protic ILs (PILs) which contains methylammonium cation and different anions like MAF, methylammonium acetate (MAAc) and methylammonium propionate (MAP). PILs have high viscosities (136 mPa s for MAAc and 94.2 mPa s for MAP) because of this film formation was initially poor. S. Öz *et al.* subsequently applied co-solvent engineering with IPA, ethanol, water, and ACN (as shown in Fig. 7), and reaching a PCE of 15% with (MA_0.15_FA_0.85_)Pb(I_0.85_Br_0.15_)_3_ as active layer, PIL/ACN as solvent system.^105^ Hoang and colleagues demonstrated a benign method to synthesize green emissive MAPbBr_3_ by using an environmentally friendly solvent based on ionic liquids. They conducted a study on a range of methylammonium carboxylate protic ionic liquids, which differed in their alkyl chain lengths. The liquids explored included methylammonium formate (MAF), methylammonium propionate (MAP), and methylammonium butyrate (MAB). Perovskite NCs with controlled sizes and shapes (nanocubes, nanorods) and subsequently high photoluminescence were obtained by controlling the alkyl chain length of the carboxylate group and reaction time.^133^ L. Gu *et al.* fabricated PSCs from perovskite ink formulations MAF, MAAc, MAP and methylammonium isobutyrate (MAIB) ILs. They studied that molecular structure of MAP led to the formation of strong Lewis adducts, providing sufficient time for large crystal formation (SEM images shown in Fig. 8a–d) resulting in solar cells with a PCE of 20.56%, also retained 88% of initial efficiency for up to 1000 hours of storage in N_2_ atmosphere.^106^ Hui *et al.* fabricated a stable α-FAPbI_3_ based PSCs using an IL, MAF. Lead iodide reacted with the MAF solvent forming N–H⋯I hydrogen bonds, which lowered the formation energy between Formamidinium iodide (FAI) and the PbI_2_ leading to a rapid transformation to the stable α-FAPbI_3_ black-phase. PbI_2_-MAF and PbI_2_-DMF : DMSO solutions and interactions in the solutions are shown in Fig. 8e. In this study, device with PCE of 24.1% in ambient air, with 93% of the initial efficiency maintained up to 500 hours as shown in Fig. 8f and g were fabricated.^104^

Chatterjee *et al.* investigated the use of ionic liquids (ILs) for synthesizing lead halide perovskite nanocrystals (NCs). They found that carboxylic acid chain length affects the emissive properties of the NCs. The researchers also discovered that lauric acid–based ILs can improve the moisture and environmental stability of the NCs. Furthermore, ILs have the potential to enhance the efficiency, stability, and synthetic toxicity of perovskite NCs.^134^ Chatterjee *et al.* utilized menthol-based deep eutectic solvents (DESs) to produce cesium lead halide (CsPbX_3_) nanocrystals (NCs) and nanoplates (NPLs) that exhibit high photoluminescence quantum yield (PLQY). These DESs possess environmentally-friendly, non-toxic, and low volatility properties, making them advantageous for use as a solvent media for the synthesis of perovskite NCs. The DESs also offer exceptional solubility for lead halide salts in the presence of oleylamine (OAm), which is critical in creating highly luminescent CsPbX_3_ NCs and 2D Ruddlesen-Popper (RP) NPLs.^135^ Zhang *et al.* used utilized natural deep eutectic solvents (NADESs) as both solvents and surface ligands for the green synthesis of cesium lead bromide perovskite nanocrystals (CsPbBr_3_). The –COOH and –OH groups in the NADESs can poly-chelate with Pb^2+^ and provide a strong interaction with CsPbBr_3_ NCs, making them suitable as both solvents and surface ligands for the NCs. The use of NADESs resulted in highly stable, bright-luminous NCs with a high photoluminescence quantum yield (PLQY) of approximately 96.8%.^136^

ILs are considered promising alternative green solvents to traditional polar aprotic solvents to produce efficient and stable PSCs. Additionally, perovskite films prepared by ILs do not require antisolvent treatments and films deposited in ambient environments are more durable, which would simplify the manufacturing of PSCs in an industrial environment. They form strong interactions with the perovskite materials, which helps to improve their stability and efficiency. ILs can provide a better environment for the perovskite materials to form more efficient films with improved performance, making them ideal solvents for this type of solar cell.

### 3.5 Solvent free deposition techniques

#### 3.5.1 Mechanochemistry

Solution-based approaches are flexible, they have limitations in terms of engineering the composition of perovskites. However, in the last decade, mechanochemistry has emerged as an eco-friendly alternative to traditional synthesis methods. Mechanochemistry is the use of mechanical force to induce chemical reactions. In perovskite solar cells, mechanochemistry can refer to the use of mechanical force, such as grinding or milling, to promote the formation of perovskite materials or to enhance their properties. This solid-state method, which involves the direct absorption of mechanical energy, is gaining popularity in organic and inorganic chemistry, as well as materials science. Mechanochemistry is a sustainable and efficient approach to synthesis that is driven by mechanical force instead of traditional wet chemical methods.^137–140^ The International Union of Pure and Applied Chemistry (IUPAC) has recently recognized mechanochemistry as one of the top 10 technologies that can have a transformative impact on the world.^141^ While mechanochemistry offers a green and efficient method for synthesizing perovskite materials, it is worth noting that conventional solvents are still required for depositing perovskite thin films.^142,143^ Although deposition techniques such as pulse laser deposition^144^ may address the issue of using conventional solvents for perovskite film deposition, it is still challenging to achieve a fully developed solvent-free PSCs.

#### 3.5.2 Melt processing

Melt processing of perovskite materials refers to a solid-state synthesis technique that involves melting the starting materials at high temperatures to form a homogeneous liquid phase, followed by quenching to form the desired perovskite phase. This approach offers several advantages, including the ability to obtain high purity materials, the elimination of the need for solvents, and the potential for producing larger single crystals with improved properties.^145^ Researchers have looked into inducing low temperature melting characteristics in 2-dimensional (2D) hybrid perovskites to be a viable deposition alternative. In this process, the precursor materials, *i.e.*, the organic amines or their halide salts and the inorganic halide salts, are solvated in aqueous hydrogen halide solution at moderate temperature (∼95 °C). Upon cooling the formed solution to room temperature, crystals of perovskites form because of supersaturation induced crystal nucleation and its subsequent growth. The obtained crystals with low melting temperatures can be melted at moderate temperatures (<200 °C) on a substrate to form a phase-pure highly crystalline as well as amorphous films.^146,147^ However, this technique is suitable for deposition of layered 2D perovskite films. For a conventional 3D perovskite for *e.g.*, MAPbI_3_, the melting temperature is higher than the temperature at which the of its degradation, hence making this technique impossible to be employed.^10^

Mechanochemistry and melt processing are emerging solid-state synthetic techniques that show great promise in the synthesis of perovskite materials. These methods offer several advantages over traditional solution-based methods, including higher reaction rates, better control over the crystal structure, reduced environmental impact, and the ability to obtain high purity materials without the need for solvents. Overall, the potential of mechanochemistry and melt processing in perovskite synthesis is tremendous and these techniques have the ability to drive new advances in materials science and technology, paving the way for the development of innovative and sustainable technologies.

### 3.6 Green antisolvents for perovskite layer engineering

Especially with the spin coating procedure, the deposition of the precursor ink is often followed by application of an anti-solvent over the film. This antisolvent aids in crystallization of the layer to yield superior perovskite film, that is conducive to achieve high efficiencies in solar cells.^107,109–112,148,149^ Antisolvent use has also been recently reported for some scalable or large area deposition techniques.^150,151^ Antisolvent treatment brings out smooth, large grain and low defect thin films of high quality. As the name suggests, the antisolvent must be a non-solvent for the perovskite and there are many solvents for this purpose. Commonly used antisolvents, like diethyl ether, chlorobenzene (CB), chloroform and toluene, have low polarity and lower boiling points than that of the solvent used to dissolve precursors.^60^ The most widely used antisolvents like halogen-based chemicals (for example, CB) are hazardous to the environment as they pollute water bodies and deplete the ozone layer. Environment-friendly alcohols like ethanol and IPA have been tested as antisolvents, but the high polarity imparted by the –OH group suppresses the growth of larger grains and defect-free perovskite films.^152^ As various solvents of low polarity exist, such toxic aromatics and chlorinated species are easily replaceable with less hazardous ones. Esters or ethers might represent good candidates and considering the efficiencies and CHEM21 solvent selection guide^33^ (Table 1), the main green solvents are ethyl acetate, methyl benzoate (MB) and anisole as well as tetraethyl orthocarbonate (TEOC).

Zhao *et al.* conducted a systematic study on the effect of volume of anisole and dripping time for formation of uniform perovskite layer over large areas.^108^ Yavari *et al.* proved the efficacy of anisole as anti-solvent by fabricating devices with PCEs of over 20%.^110^ While efficiencies greater than 20% were obtained for cells fabricated on glass/ITO substrates using anisole as antisolvent, 17% efficiency was achieved for flexible cells.^109^

**Fig. 9:**
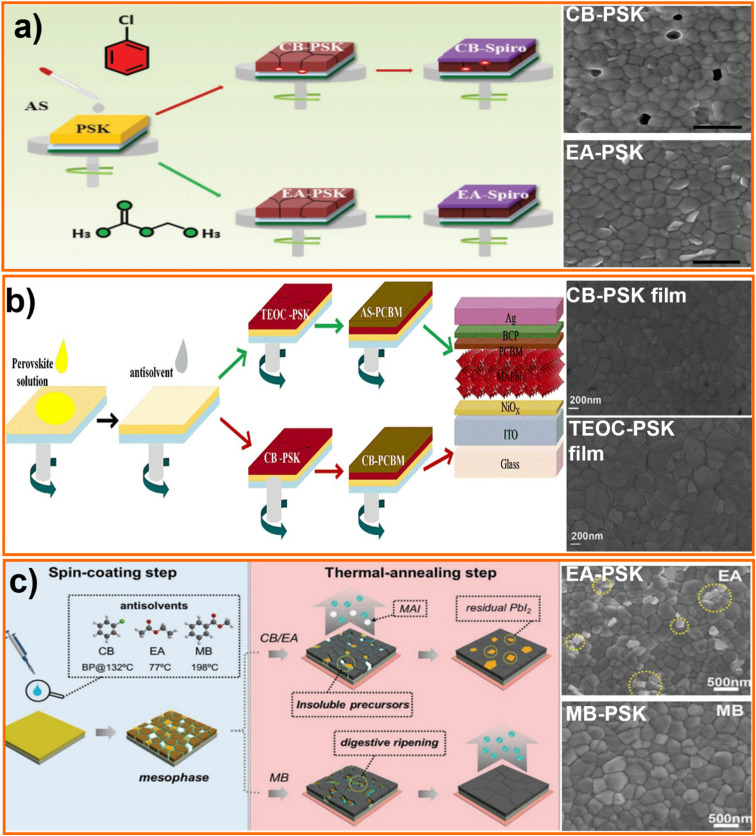
Schematic processing scheme for perovskite films treated by different antisolvents, (a) PSCs fabrication process. (a) Schematic processing scheme for perovskite films, surface SEM images of Chloro Benzene (CB) and Ethyl Acetate (EA) solvents processed perovskite films. Top view SEM images of the (a) CB-perovskite and (b) EA-perovskite films, and the scale bar is 1 μm,^112^ reproduced and copyright 2017, WILEY-VCH. (b) Tetraethyl orthocarbonate (TEOC)/anisole (AS) and chloro benzene (CB)/CB solvent systems, surface SEM images of the CB-PSK film (above) and TEOC-PSK film (below),^107^ reproduced and copyright 2020 Elsevier, (c) schematic processing scheme for perovskite films treated by different antisolvents (CB, EA, and MB). Top-view SEM images of Ethyl Acetate (EA)-PSK, and Methyl Benzoate (MB)-PSK films after annealing step^111^, reproduced and copyright 2020 WILEY-VCH.

Recently Bu group rationalized scattered literature results and reported a general approach to achieve high efficiency PSCs independently on the polarity of the antisolvent. Their study demonstrates how properly exploit mutual solvent-antisolvent miscibility, and the solubility of perovskite precursors in the antisolvent, to obtain high quality films of material. Polarity of the anti-solvent should be high enough to miscible with perovskite precursor solvent and sufficiently low to dissolve precursors with an optimum in the 2 to 4.5D range.^112^ For example, ethyl acetate has a polarity of 4.4 and a boiling point of 77 °C, which is good enough to remove the precursor solvent, providing uniform pinhole-free film (Fig. 9a) and 19.4% efficient solar cells.^112^ M. Wang *et al.* tested TEOC as a non-toxic anti-solvent, as along with the PCBM dissolved in an anisole, fabricating p–i–n PSCs with a PCE of 18.15%.^107^ As shown in Fig. 9b, the perovskite film is denser and with larger grains when applying antisolvent engineering with TEOS rather than CB reducing the density of electron trap states improving PV performance.^107^ Anisole, a frequent additive in the food and cosmetic industry, has also proven to be an effective anti-solvent.^32,108^ Tan *et al.* studied the process of creating a two-layer structured perovskite film. They began by using an antisolvent *i.e.*, ethyl acetate, on top of the first perovskite film (MAPbI_3_) to promote fast nucleation. Next, a solution of methylammonium chloride (MACI) dissolved in IPA was added to influence crystal growth, resulting in the formation of a bottom layer of MAPbI_3_ and an upper layer of mixed MAPbI_3_/MAPbI_*x*_Cl_3−*x*_. The upper layer exhibited a higher degree of crystallinity and fewer trap states due to the higher LUMO level of MAPbI_*x*_Cl_3−*x*_ in comparison to MAPbI_3_. This allowed for more efficient charge transfer without significantly impacting the overall absorption of the perovskite.^148^ Y. Yun *et al.* used a digestive-ripening agent used in the food industry called MB, to help the rapid crystallization of perovskite film and prevent the loss of organic components during the thermal-annealing step (schematic diagram shown in the Fig. 9c) to fabricate PSCs with a PCE of 22.37% and >1300 h stability.^111^ L. Xu conducted a cumulative study, analyzing the effect of CB, toluene and ethyl acetate on bromide-based perovskites and LEDs. The study demonstrated that ethyl acetate produced films with higher carriers decay time and lower defect concentrations.^153^ Ethyl acetate is also suitable as antisolvent for relatively stable cesium-based perovskites.^154^

Thus, to choose a greener antisolvent, the first and foremost property to be considered is polarity as described above. Apart from the green anti-solvents mentioned above, various alcohols, including *n*-butyl alcohol,^155^*sec*-pentyl alcohol,^156^ ethanol^157^ and IPA^150,157^ have been used. However, their performance remains lower compared to cells treated with anisole, ethyl acetate and methyl benzoate and further research needs to be carried out for alternatives. Also, most of the antisolvents explored are studied on conventional single-cation perovskite (MAPbX_3_ or FAPbX_3_ or CsPbX_3_), and there is a need to identify and greener antisolvent system for triple cation perovskite, which is capable of producing highly efficient devices. In conclusion, several green alternatives to the common halogenated and aromatic antisolvents have been reported. Among all anti solvents ethyl acetate, anisole, methyl benzoate (MB) and tetraethyl orthocarbonate (TEOC) are proven to be promising candidates.

## 4. Tackling the content of lead in the active layer

The toxicity of lead and its compounds together with the vulnerability of perovskites to humidity, heat, and light raises concerns over the commercialization of PSCs. It has been suggested that these issues can be resolved by careful device encapsulation. However, accidental damage to encapsulated solar cells is at times inevitable. Environmental fate modelling has revealed that in the case of water exposure, lead compounds can end up in water systems resulting in lead-related toxicity.^158^ Furthermore, according to Li *et al.*, perovskite contaminated soil proved to be more hazardous than other types of lead sources and it can be a threat to the food chain.^159^ The inorganic part of perovskites facilitated the absorption of lead by plants and Pb in the perovskite composition was ten times more likely to be absorbed by mint plants *via* contaminated soil. Thus, the toxicity of lead and its compounds and vulnerability of perovskites to humidity, heat, and light raises concerns over the commercialization of PSCs. Research for tackling this issue has been directed in three main directions.

Firstly, understand the true environmental and safety issues of lead, the content of which is extremely small per square meter, compared to the benefits of large-scale production of low-cost solar power, and compared to alternative power generation. No technology comes without risks. Thus, benefits and risks must be well laid out and investigated.

Secondly, devise ways to limit the escape of lead from the solar cell during its operational lifetime (especially in the event of damage) and at its end of life (*e.g.*, *via* special encapsulation/sequestration strategies).

Thirdly, design and practically fabricate solar cells with perovskite derived semiconductors that replace Pb with less problematic elements. The investigation of different lead-free perovskites to discover stable and non-toxic alternatives to lead perovskites has become a popular topic recently. The ideal lead-free perovskite suitable for commercialization must have: (i) optical and electronic properties comparable with lead-based perovskites, (ii) stability when exposed to air, moisture, light, and heat, (iii) low toxicity, and (iv) environmentally friendly fabrication routes utilizing non-toxic solvents. The emphasis of this section is to review lead sequestration techniques and then summarize lead-free PCSs in terms of efficiency, stability, and toxicity with an overview of environmentally friendly fabrication techniques of these PSCs.

### 4.1 Lead sequestration techniques

**Fig. 10:**
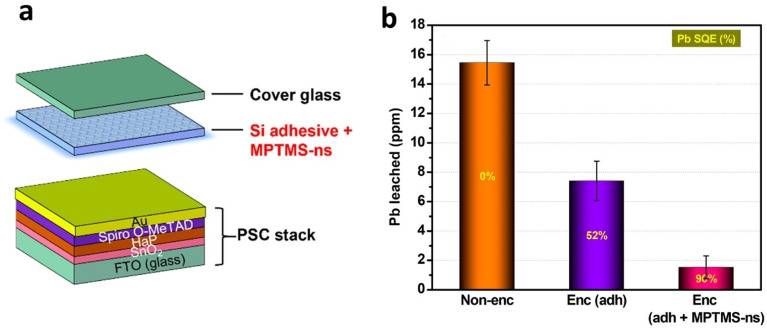
(a) Schematic of perovskite solar cell encapsulated using Si adhesive combined with lead MPTMS-ns. (b) Average Pb sequestration efficiency of non-encapsulated, encapsulated, and encapsulated + MNTMS-ns devices.^165^

The issue of lead leaching into the environment has raised concern in the scientific community and search for alternative non-toxic divalent metal cations has begun. However, the actively researched alternative, tin (Sn) shows limited degree of success due to its ambivalency and propensity towards oxidative degradation.^160,161^ Recently, such an issue has been given an out-of-box approach wherein lead can continue to be used without sacrificing photovoltaic efficiency. This unique confluence has been brought by using lead-absorbing chelating agents like transparent *P*,*P*′-di(2-ethylhexyl) methane di phosphonic acid (DMDP) and *N*,*N*,*N*′,*N*′-ethylene diamine tetrakis (methylene phosphonic acid), or EDTMP films.^162^ Such films on either side of a perovskite photovoltaic cell can offer on-device sequestration of more than 96 per cent of lead leakage caused by severe device damage (Fig. 10) due to inadvertent mechanical breakage or under harsh or acidic rainy conditions. Such techniques can offer solutions to lead leaking even at higher temperatures and heavy rain. After this first report, another group demonstrated the use of a porous metal–organic framework (MOF) polymer composite where the MOF scaffold contains a metal binding agent that stops the lead leaching.^163^ Contaminated water derived from damaged PSCs with such layer was brought below the drinkable standards set by the Environmental Protection Agency (EPA). Recently, the lead sequestration is heavily researched with multiple groups working towards turning this into a viable technology to tackle the challenge of lead leaching from PSCs using bioinspired hydroxyapatite/TiO_2_ nanoparticle (NPs) blend,^164^ thiol-functionalized NPs,^165^ and polyoxometalates-metal–organic frameworks host–guest nanostructured dopants.^166^Fig. 10a shows the schematic of an encapsulated perovskite solar cell in which the lead absorbing material, (3-mercaptopropyl)trimethoxysilane (MPTMS)-capped nanospheres (MPTMS-ns), is incorporated into the silicone-based adhesive.^165^ This low-cost thiol-functionalized delivered 90% of Pb sequestration efficiency (Fig. 10b).

### 4.2 Lead-free perovskites

**Fig. 11:**
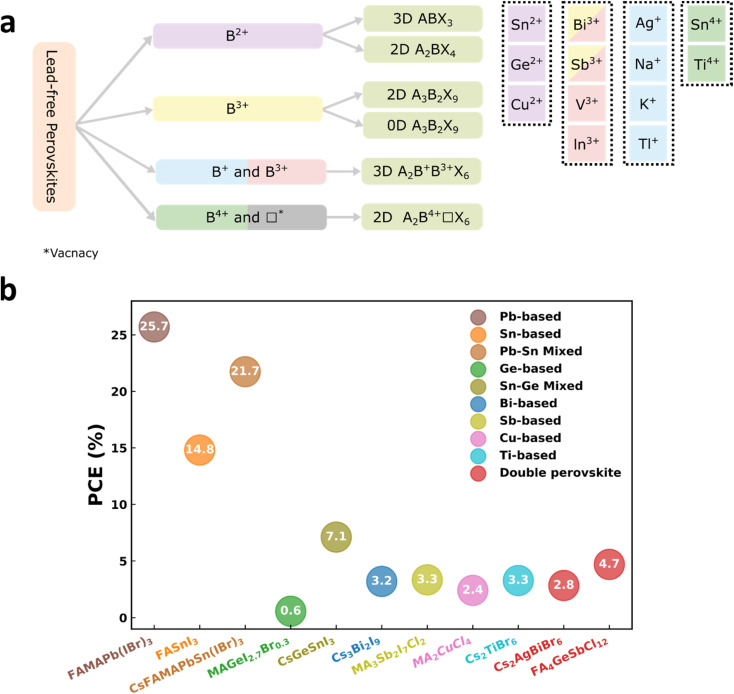
(a) Types of lead-free perovskites. (b) Efficiency record of lead-based and lead-free PSCs.^4,12,174–181^

There are a growing number of materials that have been investigated as a substitution for lead, including but not limited to group 14 elements Sn and Ge, group 15 elements Bi and Sb, and some transition metals. Depending on the ionic radius and oxidation state, perovskites form in 3D AB^2+^X_3_, 2D A_2_B^2+^X_4_, and 2D A_3_B2^3+^X_9_ phases. It is also possible to use two ion-splitting (*e.g.*, a B^3+^ and a B^+^) or ordered vacancy (*e.g.*, a B^4+^ and a vacancy) approaches to form double perovskites. In addition, some chalcogenide perovskite and perovskite-like structures are also explored as potential replacements of lead halide perovskites.^152,167^Fig. 11a shows the classification of lead-free perovskites used in solar cells and Fig. 11b. The comparison of power conversion efficiency (PCE) record of lead-based and lead-free PSCs. Perovskite PV has been shown to have remarkable efficiency indoors reaching and surpassing 30% PCEs.^168–172^ Lead free alternatives,^173^ have started to be developed for indoor light harvesting.

#### 4.2.1 Sn-based perovskites

Tin-based perovskites are the main competitor of lead-based ones as a less-toxic alternative because Sn and Pb are in the same group of elements and Sn divalent ion has similar ionic radius to that of Pb.^182,183^ Sn-based perovskites benefit from high carrier mobility and lower optical bandgaps which is more suitable for photovoltaic applications.^184^ They are the most efficient lead-free PSCs to date, achieving 14.8% PCE very recently.^180^ However, despite their significant progress, they are still far behind the 25.7% PCE record of lead-based PSCs.

**Fig. 12:**
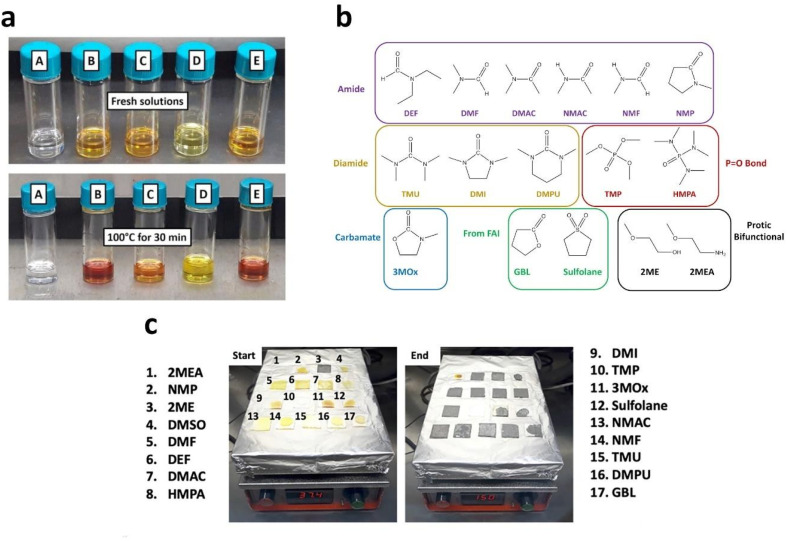
(a) Vials containing A: FAI in DMSO; B: SnI_2_ in DMSO; C: FASnI_3_ in DMF; D: FASnI_3_ in DMSO with 10 mol% SnF_2_; E: CsSnI_3_ in DMSO before and after heating at 100 °C for 30 min.^185^ (b) The 16 non-sulfoxide solvents examined for Sn-based perovskites from 6 different classes.^190^ (c) The Start and the end of drop casting FASnI_3_ solution in mentioned solvents.^190^

One of the main drawbacks of Sn-based PSCs is their stability as Sn^2+^ tends to oxidize to Sn^4+^ easily in the presence of oxygen or the widespread used DMSO solvent.^185–187^ As shown in the Fig. 12a, the color of SnI_2_ and FASnI_3_ in DMSO changes after heating at 100 °C. In addition, even the commercial batches of 99.999% SnI_2_ have found to contain considerable amounts of Sn^4+^.^188^ The Sn^4+^, which is present in the perovskite layers, acts as a p-type dopant and adversely affects efficiency by decreasing lifetime of photocarriers in the perovskite layer.^189,190^ Various approaches have been adopted to enhance stability. One is to add reducing agents such as Sn powder, SnX_2_ (X= F, Cl, Br, I, SCN), hydrazine compounds, and gallic acid to prevent Sn oxidation.^191^ However, the poor conductivity of most of these additives reduce the charge transfer in the fabricated devices. Another strategy to enhance stability is to replace the “A” cation with larger cations, to form 2D perovskites since, in 2D perovskites, organic spacing layers protect the perovskite from moisture. 2D-quasi-2D-3D Sn perovskites demonstrate superior stability by sacrificing efficiency, reaching the PCE of 9.4%.^192^ It appears that this low-dimensional perovskite compromises voltage in favour of stability as devices have the *V*_OC_ of 0.6 V compared to 0.86 V for CsSnI_3_-based cells, and other photovoltaic characteristics remain almost the same. As the stability is due to the tendency of Sn^2+^ to oxidize to Sn^4+^, one potential solution is to use Sn^4+^-based perovskites as a light absorber. Cs_2_SnI_6,_ a 2D Sn(iv)-based double perovskite is very stable and retained 95% of its efficiency after 45 days of exposure to air, yet its efficiency is limited to 2.1%.^193^ Hence, the stability issue remains unanswered to date as these perovskites are more unstable than Pb-based ones, and it is difficult to enhance their stability without sacrificing the device's efficiency.

However, when it comes to environmental impacts, the oxidation of tin to Sn^4+^ can be an advantage since when it reacts with water and oxygen, it results in the production of the inert, non-toxic SnO_2_,^194^ according to the following equations:8SnI_2_ + 4H_2_O + O_2_ → 2SnI_4_ + 6Sn(OH)I + 2HISnI_4_ + 2H_2_O → SnO_2_ + 4HI

Tin and its compounds are generally less toxic than lead compounds and are believed to be non-carcinogenic. The permissible workday exposure limit of tin is 2.0 mg in 1 m^3^ of air, 40 times higher than that of lead,^195^ and the impacts of tin in human health is insignificant compared to lead.^196^ One factor in measuring the toxicity is bioavailability, meaning to what extent living organisms absorb a material. Li and colleagues proved that mint plant tends to absorb a significantly lower amount of Sn from perovskite contaminated soil.^159^ In addition, Tin halides can potentially be more harmful to living organisms than lead halides with the same concentration in the aqueous environment.^197^ Nevertheless, the rapid oxidation of Sn^2+^ to Sn^4+^ leads to less water solubility and could reduce the bioavailability of tin. Overall, in the case of accidental leachate, tin is less likely to enter the food chain than lead.^159^

Life cycle assessment (LCA) is one of the best methodologies to evaluate the environmental impacts of solar cells from very first production stages to the end of their life. We found two studies on LCA of tin-based PSCs, with both considering a lower PCE for Sn-based solar cells compared to their current record. Zhang *et al.* assumed that MASnI_3−*x*_Br_*x*_ and MAPbI_3_ PSCs had PCEs of 5.73% and 20% respectively, while the Krebs group assumed 15.4% efficient CH_3_NH_3_PbI_3−*x*_Cl_*x*_ and 6.4% efficient MASnI_3_ solar cells.^196,198^ They concluded that when fabricating PSCs with 1 cm^2^ active area, tin-based PSCs have a lower environmental impact, energy consumption, and material consumption than lead-based ones. Considering power output, however, the environmental impacts of these PSCs change. Zhang and co-workers estimated that generating 1 kW h of electricity, tin-based PSCs emit 2.5 times higher greenhouse gasses than lead-based ones: due to their lower efficiency a larger active area is needed for tin perovskites to generate 1 kW h energy. Serrano-Lujan and colleagues concluded that to have a similar environmental footprint in large-scale production, tin-based PSCs must have at least 12% efficiency.^196^ In light of the fact that they considered a PCE of 15.4% for lead-based PSCs and the current PCE record is 25.7%, we surmise that unless tin-based PSCs surpass 20% efficiencies at the laboratory scale, they cannot be considered a strong competitor to lead-based ones. Nevertheless, it is possible to achieve promising efficiencies with Sn–Pb alloys, which are often called lead-less perovskites. For instance, Kapil and co-workers demonstrated that 21.74% efficiency is viable with tin-lead PSCs,^181^ which contain 50% less lead than conventional PSCs.

Few studies have focused on the fabrication of tin-based perovskites with non-hazardous or less hazardous solvents; typically, tin-perovskites are being deposited using DMSO or DMSO/DMF mixture. Unlike lead halides, tin halides are partially soluble in alcohol. Taking this as an advantage, Greul *et al.* fabricated lead-free perovskite solar cells based on MASnI_3_ in methanol. Methanol dissolves low concentrations of MASnI_3_ (0.1 M); still, they managed to triple the concentration by using 1,4-dioxane as a co-solvent and obtained 1.05% PCE.^13^ Although dioxane is classified as a hazardous solvent, the presence of 50% methanol is a positive step toward less-hazardous solvents in Sn-based perovskites. Another study used formic acid as a co-solvent with DMSO to fabricate FASnI_3_ solar cells with 10.37% efficiency.^199^ Formic acid is a less hazardous solvent compared to DMF and NMP, but cannot be considered as a green solvent.^200^ They suggested that acidic conditions might enhance the stability of Sn^2+^, as encapsulated devices maintained 95% of their efficiency after 200 hours of light soaking. Sn halides are soluble in acidic aqueous solutions. Abate group did a systematic solvent investigation by studying the solubility, thermal stability, and perovskite formability of FAI and SnO_2_ precursors in different solvents and found 12 non-sulfoxide solvents in which formation of FASnI_3_ occurs with no oxidation.^190^ As shown in Fig. 12b, they identified 16 non-sulfoxide solvents for FASnI_3_, by estimating solubility in 80 solvents based on HSP, dielectric constant, and calculation of the binding energy of the solvents to tin ion by density functional theory. Fig. 12c shows the drop casted films based on these 16 solvents before and after annealing comparing them with DMSO-based ink. The formation of perovskite was not observed in two of solvents and the perovskite was not stable in other two solvents after 3 hours when exposed at 100 °C. Finally, a mixture of Diethylformamide (DEF) and DMPU was selected as an optimal solvent to deposit large-grain perovskite films and a PCE of 6.2% obtained. We believe further research should also focus on the environmental impact of selected solvents for tin-based perovskites.

#### 4.2.2 Ge-based perovskites

Germanium is another member of group 14 that is viewed as a lead replacement in PSCs, but unlike tin, germanium-based PSCs are rare in the literature. This is because not only Ge(ii) has more serious stability problems than Sn(ii), but also Ge-based PSCs perform very poorly in terms of efficiency. Despite having proper bandgaps of 1.6 eV for CsGeI_3_ and 1.9 eV for MAGeI_3_, they delivered poor efficiencies of 0.11% and 0.20% respectively in first attempts.^201^ Later, MAGeI_2.7_Br_0.3_ perovskite was utilized to fabricate solar cells with 0.57% PCE obtained,^174^ which is the highest efficiency reported for Ge-based solar cells to the best of our knowledge. Both works used DMF as a solvent for deposition of Ge-based perovskites. Mixed Ge–Sn PSCs based on CsGe_0.5_Sn_0.5_I_3_ were found to be surprisingly efficient with a PCE of 7.11% and promising stability as it retained 92% of its initial efficiency after continuous operation for about 500 hours.^175^ The authors suggested that the rapid oxidation of Ge(ii) results in the formation of an ultrathin and uniform passivating layer which enhanced the stability. CsGeSnI_3_ opens new opportunities in lead-free perovskites by having superior stability to MAPbI_3_ perovskites, a suitable bandgap of 1.50 eV, and excellent optical absorption in the visible region.

**Fig. 13:**
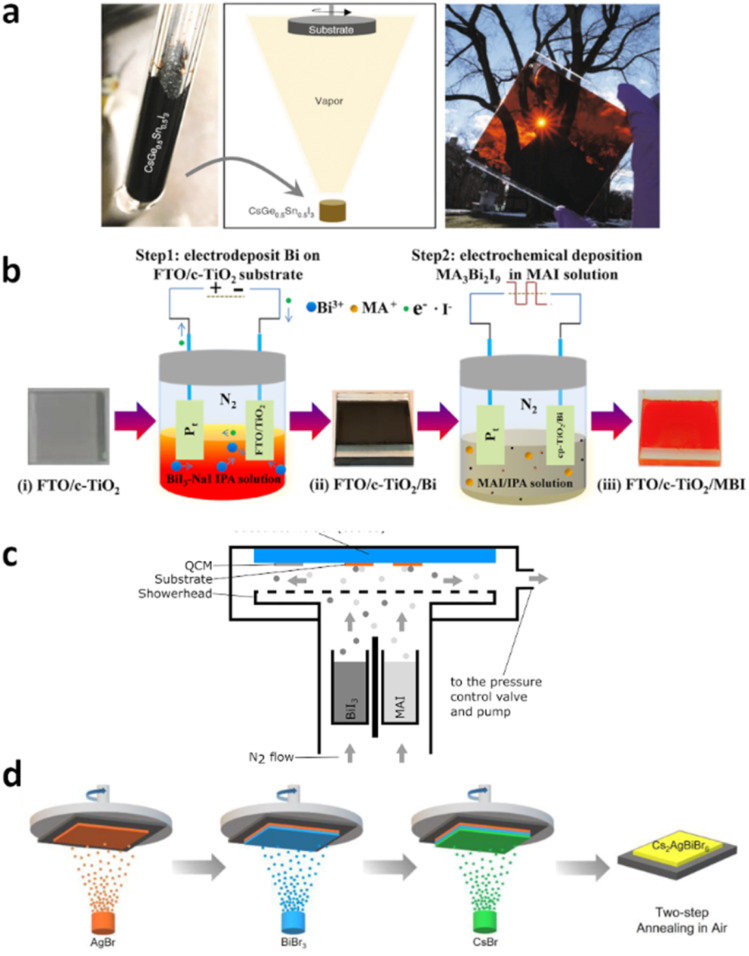
(a) Mixed Sn–Ge perovskite synthesized with melt crystallization method and deposited with thermal evaporation.^175^ (b) Schematic of two-stage electrodeposition of MBI films in isopropyl alcohol.^210^ (c) Sequential vapour deposition of Cs_2_AgBiBr_6_ double perovskites.^218^ (d) Schematic of chemical vapour deposition of Bi-based perovskites under N_2_ atmosphere.^212^

Regarding environmental impacts and toxicity, germanium is generally less harmful and less toxic than lead and its compounds. Germanium compounds are not carcinogenic, and some of its organic compounds are used for cancer treatment,^202^ but its inorganic compounds found to be damaging to the liver and lethal in some cases.^203^ However, germanium is phenomenally expensive as its price is 800 times of that of lead.^204^ This presents difficulty in producing low-cost Ge-based PSCs. It is possible to synthesize CsSnGeI_3_ by mixing CsI, SnI_2_, and GeI_2_ powders and maintaining in vacuum for 72 h at 450 °C.^175^ Solid-state reactions that eliminate solvents can be considered as an environmental-friendly fabrication route for Ge-based perovskites. It should be noted that although this method is solvent-free, the energy required can be higher than that of solution-processed synthesis methods due to prolonged exposure to high temperatures. In addition, for the fabrication of the solar cells, the obtained CsSnGeI_3_ was deposited with the energy-demanding thermal evaporation technique (Fig. 13a).

#### 4.2.3 Bi-based perovskites

Bismuth, the adjacent element to lead, has a stable trivalent ion with the same electronic configuration to Pb^2+^, the lone pair 6s^2^ state, and roughly the same ionic radius. Additionally, bismuth and its compounds are known to be relatively harmless in comparison with other heavy metals and some believed to have medicinal properties.^205^ Bi-based perovskites form in 2D structures with A_3_B_2_X_9_ formula, and larger, indirect band-gaps compared to group 14 perovskites. MA_3_Bi_2_I_9_ (MBI) has an indirect band-gap of 2.1 eV and has been reported to reach the 3.17% PCE.^206^ MBI-based solar cells showed a minor 0.1% PCE decrease after 60 days.

The primary solvents used in solution-processed Bi-based perovskites are DMF and DMSO, yet several studies focused on the fabrication of these perovskites with less hazardous solvents. One of the most recommended non-toxic solvents is ethanol, but BiI_3_ is not soluble enough in ethanol to deposit films with several hundred nanometers thicknesses that are required for solar cell absorbers.^207^ Li *et al.* managed to successfully dissolve BiI_3_ and MAI in ethanol with the addition of methylamine to the solution, and fabricate MBI-based PSCs.^208^ The single-step fabricated MBI thin-films were dense with equiaxed grains, far preferable to porous and rough films obtained by DMF solution with the same concentration. The green solvent-based MBI PSC outperformed DMF-based solar cells, delivering a PCE of 0.053% while devices with the same architecture using DMF as solvent delivered a PCE of 0.022%. These solar cells maintained their performance after 5 days of exposure to 50% humidity. Methyl-acetate (MeOAc), is another non-toxic solvent used to produce MBI thin-films, which is ranked in between recommended and problematic solvent systems.^200^ Bi-based perovskites fabricated with MeOAc demonstrated a PCE of 1.62%, the highest record among non-toxic solvent-based bismuth PSCs, and also no evidence degradation after being soaked under one sun illumination over 30 days.^209^ Korukonda *et al.* demonstrated the fabrication of pinhole free bismuth perovskite layers. Employing a greener solvent system which consists of acetone and DMF and deposition carrying out through electric-field assisted spray technique reveals the potential application of the process for eco-friendly and commercialization of Bi-based PSCs.^335^ A novel two-stage approach for deposition of MBI-films by electrodeposition in IPA bath was introduced by Want *et al.*^210^ This method did not require annealing, and after the deposition of Bi on the working electrode, the perovskite was formed in a methylammonium iodide IPA solution electrodeposition bath. Fig. 13b illustrates this two-step deposition method. Electrodeposited MBI-based solar cells have managed to reach 0.042% PCE.

Solvent-free synthesis of bismuth perovskites is also viable. El Ajjouri *et al.* demonstrated the solid-state synthesis of fifteen different A_3_Bi_2_I_9_ compounds with K^+^, Rb^+^, Cs^+^, MA and FA as “A” cation, and I^−^, Cl^−^, and Br^−^ as “X” anion by dry ball-milling of AX and BiX_3_ salts under nitrogen atmosphere.^211^ This approach opens opportunities for the green synthesis of bismuth-based perovskites by the elimination of solvents and the annealing process. However, this solid-state synthesis has not been adopted for the fabrication Bi-based of devices yet. Another solvent-free fabrication route which is widely used in semiconductor industries is chemical vapor deposition (CVD). For the first time, Sanders and colleagues fabricated MBI layers with the CVD method under an N_2_ atmosphere and total gas pressure of 1 kPa (see Fig. 13c).^212^ This method is solvent-free, requires a low vacuum, and is large-scale compatible. However, solar cells fabricated delivered only 0.016% PCE, indicating that much more optimization is required.

#### 4.2.4 Sb-based perovskites

Similar to bismuth, antimony has a stable 3^+^ ion, and its perovskites have the A_3_Sb_2_I_9_ formula and similar electronic configuration to that of group 14-based perovskites. Antimony is not only more abundant but also 30% cheaper than bismuth. However, it is not as non-toxic and environmentally-friendly as bismuth, and some of its inorganic compounds, such as Sb_2_O_3,_ are considered to be carcinogenic.^213^

MA_3_Sb_2_I_9_ (MASI) crystallizes in a 0D structure consisting of (Sb_2_I_9_)^−3^ octahedral, which are surrounded by three MA^+^ cations. Having an optical bandgap of 2.14 eV, the first attempts to fabricate PSCs based on this material resulted in 0.49% efficiency.^214^ Various strategies have been developed to synthesize 2D-structured Sb-based perovskites, including composition engineering and altering precursors. For instance, by adding chlorine, MASI transforms into its 2D phase. Recent work led by Yang *et al.* showed that uniform, pinhole-free, and low-defect MA_3_Sb_2_I_7_Cl_2_ thin-films could be obtained by incorporating bis(trifluoro methane) sulfonimide lithium (LiTFSI) additive in DMF solution of MASI perovskite.^179^ This approach led to PSCs with 3.34% PCE, the *J*_SC_ almost doubled, and devices-maintained stability after remaining 1400 hours in ambient conditions.

Just recently, the MASI synthesis form antimony(iii) acetate (Sb(OAc)_3_) precursor in different solvent systems, namely DMSO, DMF, tetrahydrothiophene-1-oxide (THTO), ethanol, and methanol has been investigated.^215^ It was shown that the 2D MASI forms in alcohol solvents, but the use of other solvents resulted in the formation of the 0D MASI phase. The 2D MASI has better stability than 0D phase. It was also revealed that methanol is a better solvent for this purpose as the solubility of Sb (OAc)_3_ is 0.25 mmol ml^−1^ in methanol, which is double the number of that of ethanol. Consequently, solar cells fabricated by methanol as a solvent for the perovskite layer perform better, achieving 0.54% efficiency. In another attempt, Zou *et al.* discovered a new group of Sb-based perovskites with (NH_4_)^+^ as A^+^ cation that are soluble in ethanol.^216^ The optical bandgap of (NH_4_)_3_Sb_2_I_9−*x*_Br_*x*_ was tuned from 2.27 eV to 2.78 eV by varying the halide in the composition. Devices based on (NH_4_)_3_Sb_2_I_9_ delivered 0.051% efficiency, with 1.03 V open-circuit voltage. Even though the use of ethanol seems promising, the authors employed hazardous CF as anti-solvent, which raises a question about the environmental impact of this method. In addition, these devices stopped working after two days of exposure to ambient air with 50% humidity.

#### 4.2.5 Cu-based perovskites

Copper is more abundant and less toxic than lead and can be a potential candidate for Pb-substitution. Copper-based perovskites form in the 2D A_2_BX_4_ phase instead of the conventional ABX_3_ perovskite structure because Cu^2+^ ion is smaller than Pb^2+^. In general, organic–inorganic Cu-based perovskites benefit from a wide tunable bandgap range (1–2.4 eV), humidity stability, heat stability, light-soaking stability, and environmentally friendly synthesis routes. However, the highest efficiency achieved by these perovskites is around 2.41% to date.^11,12^

Although there are a limited number of works on Cu-based PSCs, most of them use non-toxic solvents for synthesis and deposition of these perovskites. Cui and colleagues reported the first Cu-based perovskites used in solar cells with the chemical formula of (*p*-F–C_6_H_5_C_2_H_4_–NH_3_)_2_–CuBr_4_ and (CH_3_(CH_2_)_3_NH_3_)_2_–CuBr_4_ which have the bandgaps of 1.74 eV and 1.76 eV respectively and achieved 0.51% and 0.63% PCEs.^217^ These perovskites were synthesized in the aqueous HBr (40 wt%) and were dissolved in the non-toxic ethanol solvent for film deposition. Another Cu-based perovskite (C_6_H_5_CH_2_NH_3_)_2_CuBr_4_, synthesized, and deposited with the same method, has a bandgap of 1.81 eV, and the fabricated cells reached 0.2% PCE.^11^ This copper-based perovskite material showed high stability exposed to humidity, heat, and UV-light, the stability of devices is not reported though. Later, Elseman *et al.* demonstrated that it is possible to synthesize Cu-based perovskites by grinding precursor powders for 40 minutes.^12^ Although the perovskite powder was obtained *via* solvent-free synthesis, it was dissolved in DMF to be deposited *via* spin coating, which gave a PCE of 2.41%.

### 4.3 Double perovskites

**Fig. 14:**
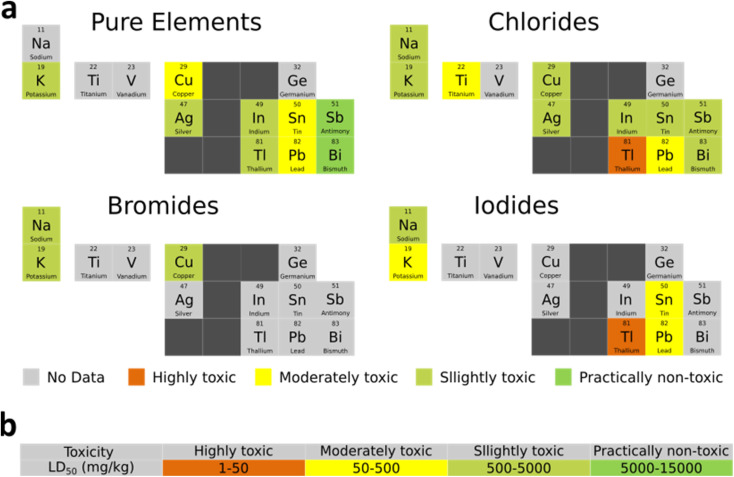
(a) The toxicity of lead and its potential replacements in perovskites and double perovskites, their chlorides, bromides, and iodides. The estimation is based on the reported LD_50_ amounts for rats in mg kg^−1^ from safety data sheets provided by Fisher Scientific. (b) The toxicity rating system of elements and compounds are based-on LD_50_ values.^226^

An additional method to synthesize perovskites is replacing 2+ cations with 1+ and 3+ cations or a 4+ cation with a vacancy (□) to form A_2_B′B′′X_6_ or A_2_B□X_6_ structure. As a result, the overall charge balance is similar to that of ABX_3_ perovskites. Examples of common ions used in double perovskites are Ag^+^, Na^+^, K^+^, Tl^+^, Sb^3+^, Bi^3+^, In^3+^, V^3+^, Ti^4+^, and Sn^4+^. These perovskites have been synthesized with both organic and inorganic A^+^ cations; the former results in the 3D structure while the latter often yields a 2D perovskite structure. Due to the wide range of materials available to produce these perovskites, the bandgap can be tuned over a wide range. Until now, the majority of discovered double perovskites had high bandgaps, and the highest PCE achieved is 4.7% by the 2D FA_4_GeSbCl_12_ perovskite.^178^ However, there are a growing number of studies in the area of double perovskite materials, and their full potential is yet to be discovered. Double perovskite powders are often synthesized with the hydrothermal method, in the acidic aqueous environments as the precursors are soluble in these solutions, but the perovskites are partially soluble in these solvent systems. For instance, only 0.05 mol of Cs_2_AgBiBr_6_ dissolved in 1 L HBr, while its solubility in DMSO was 0.6 mol L^−1^.^219^ Although there are multitudes of reports in the synthesis of double perovskites with the hydrothermal method, very few works to the best of our knowledge fabricated thin-films with non-hazardous or less hazardous solvents. However, there are reports of solid-state, solvent-free double perovskite film deposition by evaporation methods (Fig. 14d). Despite being solvent-free, evaporation techniques could be more energy-intensive than solution-processed methods as highlighted in Section 7. This section covers the current state of double perovskites in photovoltaic applications with the focus on their environmental impacts.

#### 4.3.1 Group 15-based double perovskites

Double perovskites based on group 15 elements, Bi and Sb, with the formula of A_2_B′′′X_6_ and using Ag, Ge, K, or Na as B′ metal have received much attention recently. Fabricated PSCs based on Cs_2_BiAgBr_6_ synthesized and spin-coated in DMSO, obtained 2.84% PCE.^177^ Solvent-free deposition of this perovskite have been shown by Wang *et al.* fabricated by sequential-vapour deposition of AgBr, BiBr_3_, and CsBr layers followed by annealing in the air and reached 3.7% PCE.^218^ The highest PCE record of group 15-based double perovskites is 4.7% with FA_4_GeSbCl_12_, which is a 2D Ruddlesden-Popper (RP) perovskite with *n* = 3.^178^ From the sustainability perspective, it is favorable to use sodium and potassium alkali metals as the B^+^ cation in double perovskites because their iodide salts are non-toxic food supplements, and they are very abundant elements. In fact, Na and K, each makes around 2% of the Earth's crust.^220^ However, the alkali metal-based double perovskites delivered lower performance up to now. Zhang *et al.* synthesized Cs_2_NaBiBr_6_ perovskites *via* hydrothermal approach in hydroiodic acid with the optical bandgap of 1.66 eV and good stability for up to 5 months.^221^ Devices based on this perovskite reached only 0.42% efficiency. Further optimization of perovskites is required for these double perovskites.

#### 4.3.2 Group 13-based double perovskites

There are reports of group 13-based double perovskites which have been studied as potential absorbers, but there is no report of fabricated solar cells to the best of our knowledge. For instance, indium and thallium can form ion-splitting double perovskite with silver. Lou and colleagues synthesized Cs_2_AgInCl_6_ single crystals with direct 3.2 eV bandgap which were stable under ambient conditions and elevated temperatures up to 500 °C.^222^ They proved that it is a promising material for UV photodetection. On the other hand, Tl-based double perovskite, Cs_2_AgTlX_6_, (X= Cl, Br), has a bandgap in the range of 0.95–2.0 eV which is more desirable for photovoltaic applications.^222^ Although having direct tunable bandgaps, group 13-based double perovskites are not likely to be a sustainable alternative to lead-based perovskites. Indium is a scarce and expensive metal which is not suitable for large-scale deployment, and thallium halides are extremely toxic. Thus, developing solar absorbers based on these materials would exacerbate the environmental problems of PSCs.

#### 4.3.3 Vacancy-ordered Ti-based double perovskites

The vacancy ordered double perovskites are other types of 3D lead-free perovskites with the chemical formula of A_2_B(iv)X_6_. We have already discussed Sn-based vacancy ordered perovskites in Section 4.2.1, but another exciting candidate for Pb substitution is titanium. Of the first attempts to produce Ti-based PSCs, Cs_2_TiBr_6_ is synthesized *via* sequential thermal evaporation of CsBr and TiBr_4_.^176^ The perovskite possessed a quasi-direct bandgap of 1.8 eV and carrier diffusion lengths greater than 100 nm and solar cells delivered 3.28% efficiency. Un-encapsulated devices maintained 94% of efficiency after 14 days storage at 70 °C and 30% RH. Euvrard *et al.* demonstrated that the solution-processed Cs_2_TiBr_6_ is not as stable as the evaporated one.^223^ Thus, the appropriateness of Ti-based double perovskites for photovoltaic applications remains unanswered until further investigations are conducted.

### 4.4 Lead-free perovskites; greener or not?

**Fig. 15:**
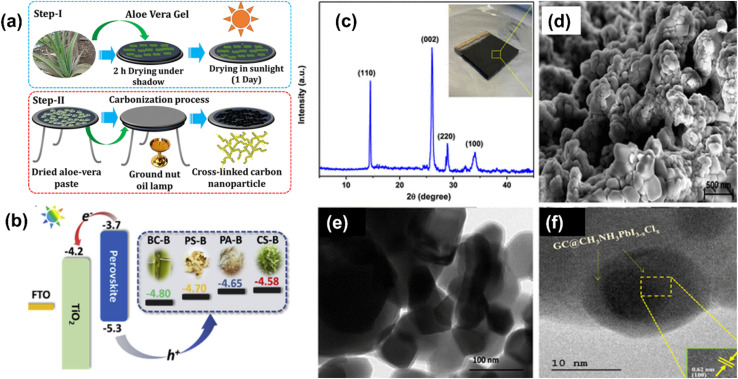
(a) Schematic representation of the preparation of aloe-vera processed cross-linked carbon nanoparticles using an ancient Indian method.^315^ (b) Energy level diagram of PSC devices based on different bio-carbon electrodes.^314^ (c)–(f) Structural and morphological properties of the bio-inspired naturally extracted graphitic carbon from an invasive plant species of *Eichhornia crassipes*. (c) XRD pattern (inset shows the digital photograph), (d) field emission microscopic SEM image, (e) high-resolution microscopic TEM image, and (f) magnified HRTEM image with lattice fringes of the deposited GC@CH_3_NH_3_PbI_3−*x*_Cl_*x*_ thin film.^317^

Table 4 and 5 summarize solvents used, deposition method, device architecture, and photovoltaic parameters of the reported lead-free PSCs in which the absorber is deposited by greener fabrication routes. This section discusses the question of the superiority or inferiority of lead-free perovskites. To address lead-free perovskites as a greener alternative to lead-based ones, several factors must be considered such as toxicity, abundance, ease of supply, cost, stability, and performance. For instance, Grancini group elaborated on lead zirconate titanate (PZT) and CdTe case studies in their perspective article on the lead-free perovskites.^225^ The most used piezoelectric material in the world, PZT, has 60% lead(ii) oxide in its compound and remained in the market so far because rival materials either lack performance, stability or competitiveness in terms of price. Similarly, CdTe solar cells are exempt from Restriction of Hazardous Substances Directive (RoHS) directives limit on the amount of Cd in consumer products. However, they could not outcompete silicon photovoltaics due to small supply of Te which restricted the annual production and consequently the market share of CdTe solar cells.^225^ One of the factors with which one can compare toxicity of elements and compounds is the oral mean lethal dose (LD_50_). Fig. 15 illustrates the toxicity of elements and halides which are used in perovskites solar cells based on the reported LD_50_. It should be noted that there are not sufficient data for measuring the toxicity of most bromide salts, and further measures to create a comprehensive toxicity database have to be taken. Based on current data, Bi and Sb could be the most non-toxic alternatives to Pb in perovskite materials. However, less toxicity alone does not make a perovskite material superior to lead-based ones. Due to its abundant supply, low price, and global production, Pb has a competitive advantage over Sb and Bi when it comes to production. Table 6 compares Pb and other candidates in terms of abundance, cost, global production, global warming potential (GWP), and whether they are listed as critical raw materials. Based on this information, tin and copper are the only viable options with relatively low GWP, low price, and large supply.

**Table 4:** Absorber material, solvents, deposition method, device architecture, and photovoltaic parameters of reported lead-free PSCs fabricated with non-hazardous or less-hazardous solvents for the perovskite layer

Absorber	Solvent	Deposition method	Device architecture	*V*_OC_ [V]	*J*_SC_ [mA cm^−2^]	FF [%]	PCE [%]	Ref.
FASnI_3_	Formic acid/DMSO (1 : 4)	Spin-coating	PEDOT:PSS/perovskite/C60/BCP/Ag	0.59	20.6	71	8.58	199
FASnI_3_	Formic acid/DMSO (1 : 1)	Spin-coating	PEDOT:PSS/perovskite/C60/BCP/Ag	0.63	22.3	74	10.37	199
FASnI_3_	Formic acid	Spin-coating	PEDOT:PSS/perovskite/C_60_/BCP/Ag	0.61	20.3	73	9.01	199
FASnI_3_	DEF : DMPU	Spin-coating	PEDOT:PSS/perovskite/C_60_/BCP/Ag	0.53	21.9	53	6.2	190
MASnI_3_	Methanol/1,4-dioxane (4 : 1)	Spin-coating	c-TiO_2_/m-TiO_2_/perovskite/spiro/Au	0.18	15.44	38	1.05	13
MA_3_Bi_2_I_9_	Ethanol	Spin-coating	c-TiO_2_/m-TiO_2_/perovskite/spiro/Au	0.84	0.17	35	0.053	208
MA_3_Bi_2_I_9_	IPA	Electrodeposition	c-TiO_2_/perovskite/spiro/Au	0.55	0.17	44	0.042	224
MA_3_Bi_2_I_9_	Methyl-acetate	Spin-coating	c-TiO_2_/m-TiO_2_/perovskite/P_3_HT/carbon	0.87	2.70	69	1.62	209
(NH_4_)_3_Sb_2_I_9_	Ethanol	Spin-coating	PEDOT:PSS/perovskite/PC_61_BM/Al	1.03	1.15	43	0.51	216
MA_3_Sb_2_I_9_	Methanol	Spin-coating	c-TiO_2_/m-TiO_2_/perovskite/spiro/Au	0.46	2.21	52	0.54	215
(*p*-F–C_6_H_5_C_2_H_4_–NH_3_)_2_–CuBr_4_	Ethanol	Spin-coating	c-TiO_2_/perovskite/spiro/Ag	0.87	1.46	40	0.51	217
(CH_3_(CH_2_)_3_NH_3_)_2_–CuBr_4_	Ethanol	Spin-coating	c-TiO_2_/perovskite/spiro/Ag	0.88	1.18	40	0.63	217
(C_6_H_5_CH_2_NH_3_)_2_CuBr_4_	Ethanol	Spin-coating	c-TiO_2_/m-TiO_2_/perovskite/P_3_HT/Au	0.68	0.73	41	0.2	11

**Table 5:** Absorber material, synthesis information, device architecture, and photovoltaic parameters of reported lead-free perovskite solar cells with solvent-free perovskite synthesis methods

Absorber	Synthesis method	Deposition method	Device architecture	*V*_OC_ [V]	*J*_SC_ [mA cm^−2^]	FF [%]	PCE [%]	Ref.
CsSnGeI_3_	Melt crystallization	Thermal evaporation	PCBM/perovskite/spiro/Au	0.63	18.61	61	7.11	175
MA_3_Bi_2_I_9_	Chemical vapor deposition		c-TiO_2_/m-TiO_2_/perovskite/spiro/Au	0.39	0.13	39	0.02	212
MA_2_CuCl_4_	Grinding milling	Spin coating in DMF	c-TiO_2_/m-TiO_2_/perovskite/spiro/Au	0.56	8.12	52	2.41	12
Cs_2_AgBiBr_6_	Sequential vapor deposition		c-TiO_2_/perovskite/P_3_HT/Au	1.12	1.79	68	1.37	218
Cs_2_TiBr_6_	Sequential vapor deposition		C_60_/TiO_2_/perovskite/P_3_HT/Au	1.02	5.69	56	3.28	176

**Table 6:** Abundance, cost, global production, Global Warming Potential, and presence in the Critical Raw Materials (CRMs) list of EU in 2020 for Pb and other elements used in lead-free PSCs^220,227–230^

Element	Abundance in Earth's crust (ppm)	Average price in 2016–2020 (USD per kg)	Global production in 2017 (tonnes)	GWP (kg CO_2_-eq. per kg)	Critical raw material for the EU in 2020
Pb	14	2.25	5 059 133	1.3	No
Sn	2.3	19.23	300 947	17.1	No
Ge	1.5	1189.60	98	170	Yes
Sb	0.2	8.42	130 751	12.9	Yes
Bi	0.0085	8.81	10 521	58.9	Yes
Ti	5650	8.31	7 027 450	8.1	Yes
Cu	60	6.03	19 939 825	2.8	No
Ag	0.075	560.64	26 469	196	No
In	0.25	374.6	790	102	Yes
Tl	0.85	7733.33	N/A	376	No

Lead-free perovskites are not likely to enter the market with their current PCE records. At the moment, the major competitor to the Pb-based perovskites, tin, achieved just a little more than half of that of Pb-based solar cells, and has problematic stability issues. Considering this, the most viable alternative is mixed Pb–Sn PSCs which can achieve more than 21% efficiency while having half amount of Pb.^181^ In the short term, the deployment of lead-less PSCs with durable and rugged encapsulation, on-device sequestration and legitimate recycling policy could be a satisfactory solution towards decreasing the environmental impacts of Pb-based solar cells. On the contrary, to completely eliminate these environmental problems, lead-free perovskites made of non-toxic elements with high stability and efficiency should be developed in the long run.

## 5. Charge transport layers

### 5.1 Solvents for the deposition of the electron transport layer (ETL)

The electron transport layer (ETL) is the least concerning material in the preparation of PSCs. For n–i–p device architectures, inorganic ETL deposition is well established. Indeed, in the most common embodiment of the n–i–p architecture, the perovskite layer is stacked on top of semiconducting oxides like TiO_2_, SnO_2_ or ZnO.^231^ Often ETLs come as a combination of a compact layer and scaffolding layer (typically mesoporous TiO_2_). Compact layers can be prepared *via* sol–gel methods starting from the appropriate organometallic precursor. Sol–gel chemistry works best when performed on mixtures of water and lower alcohols, including ethanol, IPA, and butanol. Compact layers now are of the deposited in nanocrystalline form directly, at low temperatures, compatible with flexible substrates, using water and alcohol mixtures which tend to outperform equivalent compact layers deposited from liquid precursors.^232^ Studies suggest ZnO^233^ or SnO_2_ (ref. 234 and 235) may have a less energetical impact on fabrication considering the fact that conventional TiO_2_ needs high-temperature processing. Despite consuming a large amount of energy, TiO_2_ based ETLs with ethanol as solvent are widely used due to their higher stabilities and good performance. Low-temperature-processed TiO_2_, such as the anatase processed at temperature as low as 150 °C, is an attractive option to lower embodied energies.^236^ Low temperature processing offers the benefit of fabrication of flexible devices and scaling up the technology.^60^ Light induced sintering of TiO_2_ layers is yet another energy efficient method that can be employed for plastic perovskite devices. Di Giacomo *et al.* sintered the mesoporous TiO_2_ layer first *via* UV irradiation which has been shown to get rid of solvents/binders as well as induce some sintering between nanoparticles.^237^ Different sintering procedures require different energies. A study on sintering of nanocrystalline TiO_2_ showed that the embodied energies (EE) for the processes were very high for the laboratory hotplate (37 kW h m^−2^), about half for conventional oven and belt furnace (16–18 kW h m^−2^) and down to (15 kW h m^−2^) for laser irradiation given the wall plug efficiency of the laser system was 3.5%. Obviously employing thinner substrates for conventional procedures would bring the energies down whereas designing more efficient laser systems could bring down the EE even further.^238^

In general, most oxide ETL deposition methods can be considered green. Studies revealed that ZnO and SnO_2_ processing is energy efficient and greener compared to that of TiO_2_ (ref. 239–241). In an LCA conducted over a module of 70 cm^2^ area, the energy consumption for manufacturing a TiO_2_-based module was twice compared to that of a ZnO based module as a result of the low-*T* processing of the latter (usually less than 200 °C).^239^ Similar conclusions can be determined when using SnO_2_ as ETL which can be processed at *T* < 150 °C. Najafi *et al.* reported a simple synthesis procedure of ZnO and its deposition using green solvents essentially alcohols and aqueous solutions. Interestingly, the HTL was NiO_*x*_ which is also green processed.^240^ In fact, the latter can be used in developing solar modules on PET plastic substrates.^242,243^ In another study by Gong and colleagues, two different perovskite modules with TiO_2_ and ZnO as ETLs were compared.^239^ Because they had different front and back contacts it was impossible to directly compare two ETLs however, the ZnO modules showed less energy payback time than TiO_2_ based modules. Sarialtin *et al.* performed LCA on mesoporous TiO_2_ and planar SnO_2_-based PSCs with HTM-free architecture and carbon electrodes.^241^ They concluded that using SnO_2_ as ETL, reduces the environmental impact to up to 20% compared to TiO_2_ devices (taking up 23% of the total energy required for the fabrication).

PCBM and modified PCBM are potential candidates among organic ETLs, but these fullerene-based ETLs are mostly soluble in toxic solvents like toluene and CB.^248,249^ M. Wang *et al.* showed that the greener alternative, anisole can be used to dissolve and deposit PCBM leading to devices with a PCE of 18.15%, equivalent to that with CB.^107^ Perylene diimides (PDI) based non-fullerene ETLs are another class of ETLs that are soluble in simple alcohols. Meng and co-workers studied the green solvent 2,2,2-trifluoroethanol to dissolve perylene diimides derivative(ETL).^247^Table 7 presents a summary of the photovoltaic characteristics of PSCs with different ETLs casts from a variety of solvents. SnO_2_ is currently considered a better option for low-temperature processing as it is also suitable for flexible devices and water as the solvent makes the fabrication greener.^232^

**Table 7:** Photovoltaic characteristics of ETL-based PSCs

ETL	Solvent	Device architecture	*V*_OC_ [V]	*J*_SC_ [mA cm^−2^]	FF [%]	PCE [%]	Ref.
SnO_2_	H_2_O	ITO/SnO_2_/PVSK/PEAI/spiro-OMeTAD/Au	1.16	24.90	81.40	23.56	157
SnO_2_	Ethanol	ITO/SnO_2_/PVSK/spiro-OMeTAD/Au	1.11	23.27	67.0	17.21	244
SnO_2_	H_2_O	ITO/SnO_2_/PVSK/spiro-OMeTAD/Au	1.12	23.86	80.60	21.64	245
SnO_2_	H_2_O	FTO/SnO_2_/PVSK/spiro-OMeTAD/Au	1.18	25.74	83.2	25.5	245
SnO_2_	IPA	FTO/SnO_2_/PVSK/spiro-OMeTAD/Au	1.14	21.72	76.0	19.21	246
TiO_2_	Ethanol, methoxy ethanol : terpineol = 3.5 : 1 w/w	FTO/C–TiO_2_/m-TiO_2_/PVSK/spiro-OMeTAD/Au	1.14	23.7	78.0	21.6	245
TiO_2_	Ethanol, methoxy ethanol : terpineol = 3.5 : 1 w/w	FTO/C–TiO_2_/m-TiO_2_/PVSK/spiro-OMeTAD/Au	1.14	23.30	79.6	22.7	245
PCBM	Anisole	Glass/ITO/SnO_2_/perovskite/spiro-OMeTAD/PCBM/BCP/Ag	1.06	21.9	78	18.15	107
ZnO	1-Butanol	Glass/ITO/NiO_*x*_/perovskite/PCBM/ZnO/Al	1.00	20.80	84	17.3	240
ZnO	1-Butanol	PEN/ITO/NiO_*x*_/perovskite/PCBM/ZnO/Al	1.02	20.6	73.0	15.3	240
PDIN	2,2,2-Trifluoroethanol	Glass/TIO_2_/PTAA/perovskite/PDIN/Ag	1.03	20.34	73.31	15.28	247
PDIN	2,2,2-Trifluoroethanol	Glass/TIO_2_/PTAA/perovskite/PDIN/Ag	1.09	20.75	75.09	17.00	247

### 5.2 Solvents for the deposition of the hole transport layer

Solvent requirements for the deposition of the hole transport layer (HTL) are quite different from those necessary for the deposition of the active material. In the conventional n–i–p device architecture, the HTL is deposited directly on the active layer. Therefore, the ideal solvent should dissolve the hole transport material (HTM) without affecting the underlying perovskite layer.

As HTMs are generally neutral organic molecules/polymers, HSP can be used for solubility predictions. This approach has already been applied to fullerene derivatives and organic polymers for organic solar cells,^238,250–252^ although with variable results.^251,253^ Instead, determining HSP binary *via* a solvent gradient method has proven to be a powerful tool for investigating solvent systems for OSC processing.^236,253^ Examples of application of this method to HTMs, including spiro-OMeTAD, are reported in the literature.^63,236,254–256^ The existence of software specific for HSPs calculation^41^ might make identifying solvents and solvent mixtures suitable for HTMs deposition faster, including those that are green, by expanding the solvent database.

The main factors for selecting a HTL for PSCs are: (1) an appropriate energy level to block the electrons and promote hole transport, (2) large hole mobility, even in the absence of additives/dopants (3) high solubility in non-harmful solvents.^257^

Spiro-OMeTAD has historically been the most popular HTL used in conventional n–i–p architectures delivering efficient devices, with PCEs of up to 25.5%.^258^ Spiro-OMeTAD and similar related molecules are typically cast from chlorinated and highly aromatic solvents such as CB and DCB or in toluene which are toxic. Nevertheless, aromatic solvents with a moderately green profile exist. Among bio-renewable aromatic solvents displaying low toxicity, *p*-xylene and anisole have been successfully employed for PSC fabrication. Isabelli *et al.* in particular used the former for HTL processing, producing devices having PCE of ∼14%, essentially matching the values obtained from analogous devices processed with CB.^255^ Jiang *et al.* demonstrated that THF can be used instead of CB to obtain efficient devices (PCE of 17%),^259^ thus other greener ethers (such as 2-MeTHF, or 2-MA) can be studied for the deposition of spiro-OMeTAD HTL. In 2022, L. Beverina group synthesized spiro-OMeTAD by green processing route, the performance of the new spiro showed equal performance as commercial spiro-OMeTAD. It significantly reduced the overall *E*-factor a green chemistry metric measuring the synthesis's waste/purified product ratio, from 5299 to 555, eliminating chlorinated solvents and hazardous chemicals in the synthesis process.^260^

LCA studies on HTMs are limited to considering spiro-OMeTAD and CuSCN for n–i–p structures, and PEDOT:PSS for p–i–n cells. Maranghi *et al.* reported six different LCA studies with the cradle-to-gate approach concluding the environmental impacts of different device architectures and deposition processes.^261^ They realized that the environment impact of the CuSCN is smaller than that of spiro-OMeTAD. The main contributor to the high environmental impacts of spiro-OMeTAD is the energy consumption during the deposition. Furthermore, the tedious synthesis procedure followed by rigorous purification makes it expensive. Also, spiro-OMeTAD is unstable when exposed to the ambient atmosphere. This has triggered the need for identifying substitutes for spiro-OMeTAD. There are a growing number of HTMs used in PSCs, as can be seen in the following sections. The use of non-hazardous solvents for organic HTMs deposited over the perovskite layer is rarely reported. The challenging task for this is to identify solvents with which the underlying perovskite film remains undisturbed if not even aiding its crystallization. At the moment, significant variations in batch-to-batch production and lower number of purification steps required as compared to polymers promote the use of small molecules as HTMs.^262^ Nevertheless, more LCA studies are required to compare the environmental impact of different HTMs.

#### 5.2.1 Small molecule HTMs

Tetrahydrofuran (THF) is a non-halogenated, non-aromatic alternative to CB and toluene, which effectively dissolves HTMs, besides being also used as additive to form a superior absorber layer.^263–265^ Wu *et al.* determined that a smaller amount of spiro-OMeTAD is required when cast from THF rather than from CB.^266^ The same group also synthesized new small molecules dissolved in THF with efficiencies of around 18%.^266^ Tetraphenylethylene (TPE)-based small molecules, both fused and non-fused, can be considered valid alternatives to spiro-OMeTAD since they can be processed advantageously in THF.^267^ The TPE-derivative with -NMe substituent showed better solubility (in CB) in comparison with the TPE based molecule (with a solubility of 40 mg ml^−1^*vs.* 15 mg ml^−1^).^268^ However, synthesis and processing of these TPE-based HTMs involve toxic and hazardous solvents like toluene and CB apart from the need for additives to improve hole mobility of the material.^269^ As a recommendation, MeTHF, anisole, *p*-xylene and 2-methyl anisole should be explored in the future as greener alternatives to THF.

#### 5.2.2 Polymer HTMs

Poly(3,4-ethylenedioxythiophene):poly(styrene sulfonate) (PEDOT:PSS) was initially developed for OLEDs,^270,271^ then incorporated into organic solar cells,^272^ and has since become a common HTL for p–i–n perovskite solar cells.^273^ Whereas PEDOT is insoluble in water, addition of PSS as a polymer surfactant, enables the dispersion of PEDOT:PSS in water. Most commercial PEDOT:PSS formulations are sold as aqueous inks.^273^ Although versatile, easy to use and deposit over large areas questions marks remain for this material as a result of limited electron blocking capabilities^273,274^ to reach the very highest efficiencies and the hygroscopicity of PEDOT:PSS which affects stability of the perovskite cell stack.^275^

2-MA, which is used as food additives, is an excellent solvent for modified polymers with benzo[1,2-*b*:4,5:*b*′]dithiophene (BDT) backbone. Lee *et al.* synthesized asymmetric benzothiadiazole (BT) and BDT based polymer (asy-PBTBDT) which is highly soluble in 2-MA and displayed similar performances to conventional spiro-OMeTAD for a meso-porous n–i–p PSC.^257^ Also asy-PBTBDT with higher molecular weight (MW) exhibited better solubility and superior properties than lower MW asy-PBTBDT in 2-MA solvent.^276^ BDT based polymers with lead capturing ability due to presence of alkoxy-tetraethylene glycol chains (also improving their solubility in the green solvents 2-MA and 3-methylcyclohexanone (3-MC)) triggered development of lead leakage from perovskite lattice green fabrication systems.^277^ Thiophene based P3HT is extensively used in organic photovoltaics, owing to its ease of processability and tunable optical properties *via* varying side chains and or molecular weight.^278,279^ Recently, Jeong *et al.* identified gallium acetylacetonate-doped P_3_HT as HTL to improve the stability of the device (which remained stable for 2000 hours at RH of 85%). Although the efficiencies are reported to be 22.9% and 24.6% for undoped and doped P_3_HT HTL based devices, use of CB as solvent for P_3_HT is concerning and has to be addressed.^280^Table 8 provides a concise overview of HTMs, solvent choices, device architectures, and key photovoltaic parameters for perovskite solar cells (PSCs).

**Table 8:** Photovoltaic characteristics of HTL-based PSCs

HTL	Solvent	Device architecture	*V*_OC_ [V]	*J*_SC_ [mA cm^−2^]	FF [%]	PCE [%]	Ref.
TPE-NMe	CB	FTO/TiO_2_/CH_3_NH_3_PbI_3−*x*_Cl_*x*_/HTM/Ag	0.87	21.69	73	13.78	268
TPE-4DPA	CB	FTO/TiO_2_/CH_3_NH_3_PbI_3−*x*_Cl_*x*_/HTM/Ag	1.02	19.63	0.65	13.04	268
Dopant-free spiro	CB	ITO/C60/MAPbI_3−*x*_Cl_*x*_/dopant-free spiro/MoO_3_/Ag	0.99	20.87	73.83	15.27	266
Dopant-free spiro	THF	ITO/C60/MAPbI_3−*x*_Cl_*x*_/dopant-free spiro/MoO_3_/Ag	1.02	21.29	77.78	16.94	266
Po-TPE-4DPA	CB	FTO/TiO_2_/CH_3_NH_3_PbI_3−*x*_Cl_*x*_/HTM/Ag	1.02	19.23	41	8.08	267
Pm-TPE-4DPA	CB	FTO/TiO_2_/CH_3_NH_3_PbI_3−*x*_Cl_*x*_/HTM/Ag	1.07	20.04	72	15.44	267
Pp-TPE-4DPA	CB	FTO/TiO_2_/CH_3_NH_3_PbI_3−*x*_Cl_*x*_/HTM/Ag	1.03	19.63	65	13.04	267
CJ-01	CB	FTO/TiO_2_/perovskite/HTM/Au	1.11	22.32	74.7	18.56	269
CJ-02	CB	FTO/TiO_2_/perovskite/HTM/Au	1.06	18.5	63.5	12.5	269
Spiro	CB	FTO/TiO_2_/perovskite/HTM/Au	1.08	22.72	76.0	18.69	269
Di-TPA	Toluene	FTO/TiO_2_/MAPbI_3_/HTM/Au	1.03	20.5	73.3	15.5	281
Tri-TPA	Toluene	FTO/TiO_2_/MAPbI_3_/HTM/Au	1.03	21.4	74.4	16.4	281
Tetra-TPA	Toluene	FTO/TiO_2_/MAPbI_3_/HTM/Au	1.05	22.0	78.0	18.0	281
CuGaO_2_	Iso-proponal	FTO/c-TiO_2_/perovskite/CuGaO_2_	1.11	21.66	77	18.51	282
Spiro	CB	FTO/c-TiO_2_/perovskite/spiro	1.08	21.45	74	17.14	282
DPIE	—	FTO/bl-TiO_2_/mp-TiO_2_/CH_3_NH_3_PbI_3_/HTL/Au	0.85	15.8	58	7.75	283
Dopant free spiro-MeOTAD	—	FTO/bl-TiO_2_/mp-TiO_2_/CH_3_NH_3_PbI_3_/HTL/Au	0.86	14.9	61	7.87	283
DPIO	—	FTO/bl-TiO_2_/mp-TiO_2_/CH_3_NH_3_PbI_3_/HTL/Au	0.96	15.08	70	10.14	283
asy-PBTBDT	2-MA	FTO/TiO_2_/m-TiO_2_/perovskite/HTM/Au	1.14	22.4	73.2	18.2	276
asy-PBTBDT	2-MA	ITO/PEDOT:PSS/perovskite/asy-PBTBDT/Au	1.11	22.4	73.2	18.3	257
Spiro-OMeTAD	CB	ITO/PEDOT:PSS/perovskite/spiro/Au	1.11	22.1	74.9	18.5	257
Alkoxy-PTEG	2-MA	FTO/SnO_2_/perovskite/alkoxy-PTEG/Au	1.14	23.2	79.8	21.2	277
Alkoxy-PTEG	3-MC	FTO/SnO_2_/perovskite/spiro-OMeTAD/Au	1.13	23.3	75.7	19.9	277
Spiro-OMeTAD	CB	FTO/SnO_2_/perovskite/spiro-OMeTAD/Au	1.13	23.3	78.3	20.6	277
CuSCN	Propyl-sulfide	FTO/TiO_2_/CH_3_NH_3_PbI_3_/CuSCN/Au	1.02	19.2	58	11.4	284
CuSCN	Propyl-sulfide	FTO/bl-TiO_2_/m-TiO_2_/CH_3_NH_3_PbI_3_/CuSCN/Au	0.92	18.7	56	9.79	285
CuSCN	Propylsulfide + IPA (1 : 2) + MAI (10 mg ml^−1^)	FTO/bl-TiO_2_/m-TiO_2_/CH_3_NH_3_PbI_3_/CuSCN/Au	0.92	19.4	56	10.07	285
CuI	ACN	FTO/TiO_2_/CH_3_NH_3_PbI_3_/CuI/Au	0.73	32.72	31	7.4	286
Cu_2_O	Evaporation	FTO/TiO_2_/CH_3_NH_3_PbI_3−*x*_Cl_*x*_/Cu_2_O/Au	15.8	0.96	59	8.93	286
Spiro-OMeTAD	CB	FTO/TiO_2_/CH_3_NH_3_PbI_3−*x*_Cl_*x*_/spiro-OMeTAD/Au	17.0	0.99	68	11.5	286
PEDOT:PS	CB	FTO/PEDOT:PS/CH_3_NH_3_PbI_3_/PCBM/BCP/Ag	0.91	20.0	81.0	14.8	287
Spiro-OMeTAD	THF	Glass/ITO/C_60_/MAPbI_3−*x*_Cl_*x*_/spiro-OMeTAD/MoO_3_/Ag	1.023	21.29	77.78	16.94	266
NiO_*x*_	H_2_O	Glass/ITO/NiO_*x*_/perovskite/PCBM/ZnO/Al	1.00	20.80	84	17.3	240
NiO_*x*_	H_2_O	PEN/ITO/NiO_*x*_/perovskite/PCBM/ZnO/Al	1.02	20.6	73.0	15.3	240
NiO_*x*_	IPA	ITO/NiO_*x*_/DEA/CH_3_NH_3_PbI_3_/C60(CH_2_)(Ind)/PN4N/Ag	1.13	20.4	80.0	18.1	288
NiO_*x*_	—	Glass/ITO/NiO_*x*_@NaCl/perovskite/PCBM/ZrAcac	1.14	22.83	79.6	20.71	288
NiO_*x*_	—	Glass/ITO/NiO_*x*_@KCl/perovskite/PCBM/ZrAcac	1.15	22.89	79.5	20.96	288
NiO_*x*_	EtOH	ITO/NiO_*x*_/MSs/perovskite/PC61BM/BCP/Ag	1.12	22.34	80.8	20.34	289
PTAA	Toluene	Glass/FTO/C–TiO_2_/m-TiO_2_/PVSK/PTAA/Au	0.91	19.30	70.20	12.3	290
PTAA	Toluene	Glass/FTO/C–TiO_2_/m-TiO_2_/PVSK/PTAA/Au	0.99	16.5	72.7	12.0	291
PTAA	Toluene	Glass/FTO/C–TiO_2_/m-TiO_2_/PVSK/PTAA/Au	1.06	24.7	77.50	20.1	292

#### 5.2.3 Inorganic HTMs

So far, organic HTMs have been the most-used materials for fabricating highly efficient devices, at least in the n–i–p architecture. But, as discussed, these HTMs are generally processed using toxic and hazardous solvents and additives are incorporated to increase conductivity. Inorganic copper based HTMs such as CuI, CuO, CuSCN, CuO_2_ are all solution processable and form a uniform layer. CuSCN, as HTM for either n–i–p or p–i–n configuration, is solution processable using di-propyl sulfide or propyl sulfide.^284,285^ It is worth noting these solvents are found in food items like garlic, onion and mustard and food additives too.^284^ Furthermore, using an aqueous solution of copper salts is another alternative to deposit CuSCN *via* electro-deposition. Although CuI as HTM is usually processed in a CB solution it can be dissolved in ACN and di-propyl sulphide as well as being simply thermally evaporated.^286^

Copper oxides are yet another p-type semiconductor, mostly used in p–i–n PSCs with organic ETLs. A report suggested solvent free sputtering for deposition of high quality Cu_2_O as HTM. The device delivered an efficiency of 9%.^293^ When it comes to solution processing, aqueous solutions of nitrate/iodine/copper are coated followed by diluted NaOH treatment and methanol wash.^287,294^ Sun *et al.* achieved a PCE of 17.1% in PSCs by optimizing the spin-coating deposition of CuO_*x*_ film using 1,2-dichlorobenzene as the solvent, suggesting potential for further improvement.^295^

Nickel oxide is a versatile inorganic HTM for PSCs owing to its flexibility in being deposited with either chemical or physical methods. Similarly, to CuO_*x*_, NiO_*x*_ is obtained by reacting nitrates, acetates, or halide salts of nickel with NaOH or certain amines in polar solvents like water or ethanol. Once the process yields NiO_*x*_ nanoparticles, the particles are dispersed in suitable green solvents, usually water of ethanol and are either spin-coated or printed. Otherwise, electro-deposition, RF sputtering, e-beam evaporator, pulsed laser deposition (PLD) are alternative advanced deposition techniques for NiO_*x*_ that have been reported. In p–i–n solar cells, with NiO_*x*_ as HTL, efficiencies of over 20% have been reached.^289,292,296^ Xie *et al.* spray coated a nickel precursor in 1 : 1 ACN and ethanol, to manufacture a FTO/NiO_*x*_/FAPbI_3_/PCBM/TiO_*x*_/Ag device with a PCE of 20%.^297^ Chang *et al.* fabricated a PSC with a low-cost interface layer material (18–22 USD per g), MS-OC in NiO_*x*_ based HTL gave a promising efficiency of 20.34%.^289^

In summary, although dopant-free organic HTMs have attracted more attention in recent years, researchers have rarely reported processing these materials in green solvents. As an alternative, inorganic HTMs offer advantages of choice of processing, deposition, and solvents. NiO_*x*_ based inorganic HTL is a good choice among all other HTLs regarding its stability and green processing.

## 6. Electrode materials

Selection criteria for top electrodes in perovskite solar cells generally focus on electrical conductivity and stability.^298^ For industrialization purposes cost and sustainability become also fundamental.

### 6.1 Top metallic electrodes

Au can still be considered as the metal of choice for PSCs. Considering the environmental impact of acquiring, processing and discarding (if not recovered) the metal, makes the use of Au a concern.^299^ In fact, a LCA study carried out by Gong and co-workers revealed that gold electrodes contribute to more than half of both prime energy consumption and carbon footprint of raw material production of perovskite solar modules.^239^ The extraction and purification process of gold requires a great deal of energy and hence is expensive. Moreover, the process involves toxic chemicals such as mercury and cyanide.^300^ It has been shown that Au can be replaced by other metals such as Ag or Al, which would reduce energy consumption related to the material but are far from ideal choice especially because they are known to diffuse through the transport layers and interact with the perovskite semiconductor and lead to its degradation. In p–i–n, people often use bathocuproine (BCP) to avoid metals diffusion and also BCP cast from the green solvents.^301^ Introduction of MoO_*x*_/Al as back contact has resulted in around a 9% reduction in energy demand as well as global warming potential when compared to silver.^302^ Alternative choices for metallic top electrodes such as Cu, Ni, W, and Mo, have been explored by Wang *et al.*^298^ These materials were deposited by sputter coating and the energy requirement for these materials is less in comparison with metals discussed earlier. There have been notable attempts to identify a stable top electrode material that can be processed at lower energy.

#### 6.1.1 Carbon electrodes

Carbon based electrodes have potential as electrode materials that address industrial, stability and environmental concerns. Furthermore they also prevent migration of halogen ions^303^ which is a well-known issue with metallic electrodes.^304^ Additionally, the hydrophobic nature of carbon electrodes shields the perovskite layer and protects the cell against moisture, which makes it an additional intrinsic layer of encapsulation to the device.^305^ Carbon in various forms, including carbon black, carbon nanotubes, both single and multi-walled, graphite, reduced graphene oxide *etc.*, have been tested as electrodes. Scalable deposition techniques such as blade coating or screen printing have been used for the deposition of these electrodes.^306^ CNTs can be synthesized using sustainable Floating-catalyst (FC) aerosol CVD and incorporated in transport layers.^307^ The solvents and binders used for processing carbon materials like-ethanol,^308^ ethyl acetate,^309^ terpineol, cellulose,^310^ organic acetates,^309^ IPA^311^ are non-toxic and non-hazardous in nature.

Carbon black, graphite/amorphous carbon, graphene, and carbon nanotubes are the most frequently used carbon materials in carbon-PSCs, though their preparation processes can be complex and expensive. As a result, bio-based carbon derived from various types of biomass waste has emerged as a promising carbon catalyst due to its abundance and ease of accessibility. In 2014 the porous carbon with a high surface area, which is prepared by pyrolysis and chemical activation of rice husk, is being examined as a counter electrode in dye-sensitized solar cells by Wang *et al.* A dye-sensitized solar cells (DSSCs) with this porous carbon counter electrode exhibited a conversion efficiency of 6.32%, which is similar to that of a cell with a Pt electrode (6.69%).^312^ These encouraging results with rice huck porous carbon opened a new door to apply a potential electrode for DSSCs. Later in 2017 Yu *et al.* created bio-based porous carbon from quince leaves as a catalyst for DSSC fabrication and achieved a high PCE of 5.52%.^313^

The first report on the use of bio-based carbon as electrodes in PSCs (perovskite solar cells) was published by Mali *et al.* in 2018.^315^ They introduced an aloe-vera processed carbon electrode made from naturally extracted cross-linked carbon nanoparticles, traditionally used in ancient Indian process, for mesoscopic perovskite solar cells. In the experiment as shown the Fig. 15a, the aloe vera gel was first extracted from the leaves and dried in sunlight for one day (Step I). Next, the dried gel was heated overnight using an oil lamp (Step II), which converted it into a black powder. The black powder was then scraped from a SS (stainless steel) substrate and ground using a ball mill. The fine activated carbon (AV-C) powder was washed with 2 M HCl and annealed in an argon atmosphere at 1000 °C. The carbon paste was coated by mixing the powder with chlorobenzene solvent (1 : 2 wt%/vol%) using screen-printing technique. Resulting more than 1000 hours stable PSC with PCE of 12.58%. In 2020 Gao *et al.* prepared bio-carbons using four different types of biomasses bamboo chopsticks (BC-B), peanut shell bio-carbon (PS-B), corn stalk (CS-B), and phragmites australis (PA-B) bio-carbons have been selected.^314^ It has been observed that the open-circuit voltage (*V*_oc_) loss in the *J*–*V* curve was primarily determined by the energy level alignment between PVK and bio-carbon CEs, as shown in Fig. 15b as a result *V*_oc_ of the devices decreased with the energy level mismatch. The PSCs based on BC-B CEs had the optimal energy band and highest *V*_oc_ when compared to the others. Which resulting ultra-low cost shown in Table 9 bio-carbon materials are significantly cheaper than other commercially available carbon materials and cost compared to other carbon-based materials as they are three orders of magnitude cheaper than the least expensive carbon material. Which resulting ultra-low cost BC-B CEs with 2000 hours stable PCE of 12.82%. In 2022 Xie *et al.* reported, a N, O co-doped porous composite carbon electrode was prepared using a spraying method. The electrode, named N-KSDC, was made from KOH-activated soybean dregs, conductive carbon black, and polymethylmethacrylate.^316^ This method was employed to enhance the interface qualities between the perovskite and carbon and improve the efficiency and stability of C–PSCs. The best power conversion efficiencies of N-KSDC-based C–PSCs were 13.45% and 11.08% for active areas of 0.08 cm^2^ and 1 cm^2^, respectively.

**Table 9:** Price of different carbon electrode materials for PSCs.^314^

Materials	Supplier	Price ($ per kg)
Graphene	Sigma-Aldrich	1 271 660.01
Carbon nanotubes	XFNano	44 030.38
Fullerene-C60	Alfa Aesar	421 204.57
Fullerene-C70	Alfa Aesar	1 929 026.52
Carbon paste	DYCOTEC	399.10^b^
Carbon black	Alfa Aesar	227.15
Graphite powder	Sigma-Aldrich	78.16
Bio-carbon	Waste recycling	0.49^a^

a The corresponding calculation of bio-carbon cost was shown in ref. 314.

b Carbon paste from DYCOTEC added to original table.

Pitchaiya *et al.* used graphitic carbon extracted from a plant species called *Eichhornia crassipes*, which served as both hole transporter and top electrode for the device.^317^ Properties of graphitic carbon extracted from the invasive plant species *Eichhornia crassipes* shown Fig. 15c that the XRD analysis of the graphitic carbon-encapsulated perovskite thin film (GC@CH_3_NH_3_PbI_3−*x*_Cl_*x*_) revealed the presence of tetragonal perovskite structure with peaks at 2*θ* = 13.97°, 28.32°, 31.78°, and 40.39°, and hexagonal graphitic carbon at 2*θ* = 26.12°, seen in the apical sites of the perovskite structure. Fig. 15d The FESEM image in Fig. 1b reveals a coagulated island-like structure, which is likely the result of strong coordination between Pb^2+^ ions and the C=O group found in porous graphitic carbon. This observation supports the formation mechanism of the GC@CH_3_NH_3_PbI_3−*x*_Cl_*x*_ structure that is encapsulated. Fig. 15e and f high-resolution TEM images reveal perovskite crystals in a darker region, which are trapped inside capsules of graphitic carbon (appearing in lighter contrast). Resulting devices shown 20% PCE under 200 lux of light and device also had a maximum PCE of 6.32% under 1 Sun illumination.

In conclusion, various bio-based carbon materials have been successfully prepared from biomass waste such as aloe-vera peel, fallen quince leaves, rice husk, bamboo chopsticks, peanut shell bio-carbon, corn stalk, and phragmites australis, *Eichhornia crassipes*, and soybean dregs. These bio-based carbon materials have been effectively utilized as superior ultra-low cost counter electrode catalysts for DSSCs and PSCs. The stability of bio-carbon based perovskite solar cells is an active area of research. However, initial studies have shown promising results in terms of stability, with some bio-carbon based perovskite solar cells showing good stability under various environmental conditions such as high humidity and heat. However, long-term stability and durability of these cells still need to be studied more extensively. Additionally, the stability of bio-carbon based perovskite solar cells also depend on the method of preparation, properties of bio-carbon materials, and device architecture. Therefore, more research is needed to fully understand the stability of bio-carbon based perovskite solar cells and to improve their performance and durability. The PCE of bio-carbon based perovskite solar cells is an active area of research. Bio-carbon based perovskite solar cells have shown promising results in terms of PCE. Above studies have reported PCE reach nearly 13%, however, it's worth to mention that the PCE of bio-carbon based perovskite solar cells can vary depending on the method of preparation, properties of bio-carbon materials, and device architecture. Additionally, it's important to note that the PCE is not the only metric to evaluate the performance of a solar cell, other parameters such as stability, durability, cost, and scalability also play an important role. Therefore, more research is needed to improve the PCE, and overall performance of bio-carbon based perovskite solar cells.

#### 6.1.2 Front transparent contacts

According to the life cycle assessment of a typical perovskite solar cell with glass/FTO/TiO_2_/spiro-OMeTAD/Au architecture, FTO preparation accounts for 28% of device fabrication energy, and 44% of embedded material energy.^318^ The deposition and etching of fluorine-doped tin oxide (FTO) and indium-doped tin oxide (ITO) are energy-demanding. In addition to that the ultrasonic cleaning of the substrate consumes a large amount of solvents. Besides, the presence of tin in ITO and FTO presents toxicity and supply concerns.^319^ It has also been demonstrated that ITO consumes 2.5 times more energy than FTO due to the presence of energy-intensive indium.^239^ This necessitates the replacement of ITO/FTO front contacts with greener alternatives. To the best of our knowledge, there is no LCA study using aluminum-doped zinc oxide (AZO) as front contact, but it is a prospective replacement having less environmental impact.^320^ Glass is responsible for around 98% of the total mass of perovskite solar cell because of its relatively higher thickness compared to other layers.^239^ To reduce the embedded energy of the substrate, alternatives such as paper and cellulose-based substrates,^321,322^ or ultra-thin glass^323^ can be developed. For instance, roll-to-roll ITO-coated ultra-thin glass with a thickness of only 100 microns was used to fabricate PSCs with best PCEs under indoor illumination.^323^

## 7. General directions from life cycle assessment of PSCs

Life cycle assessment (LCA) is a standard procedure to estimate and assess the environmental impacts of manufactured products, processes, or services throughout their lifetime.^45^ We have included LCA information for the different layers used in perovskite cell fabrication. Here we touch open broader aspects. In the assessments of devices like solar cells, environmental impacts can be evaluated from the first stage of raw material extraction and processing (cradle) to the manufacturing stage, consumer usage, and finally, the disposal or recycling stage (grave). The environmental footprints are estimated by considering different factors such as greenhouse gas emissions, consumed energy, used materials, waste, human toxicity, environmental toxicity, *etc.* Functional units such as kW h of generated electricity or constant module area are used to provide consistent comparisons of solar modules. Several studies performed LCA on PSCs and modules.^158,196,239,302,324–330^ These studies can be helpful in the comparison of different perovskite device architectures as well as perovskite modules with other photovoltaic technologies to point researchers toward more environmentally friendly solutions.

**Fig. 16:**
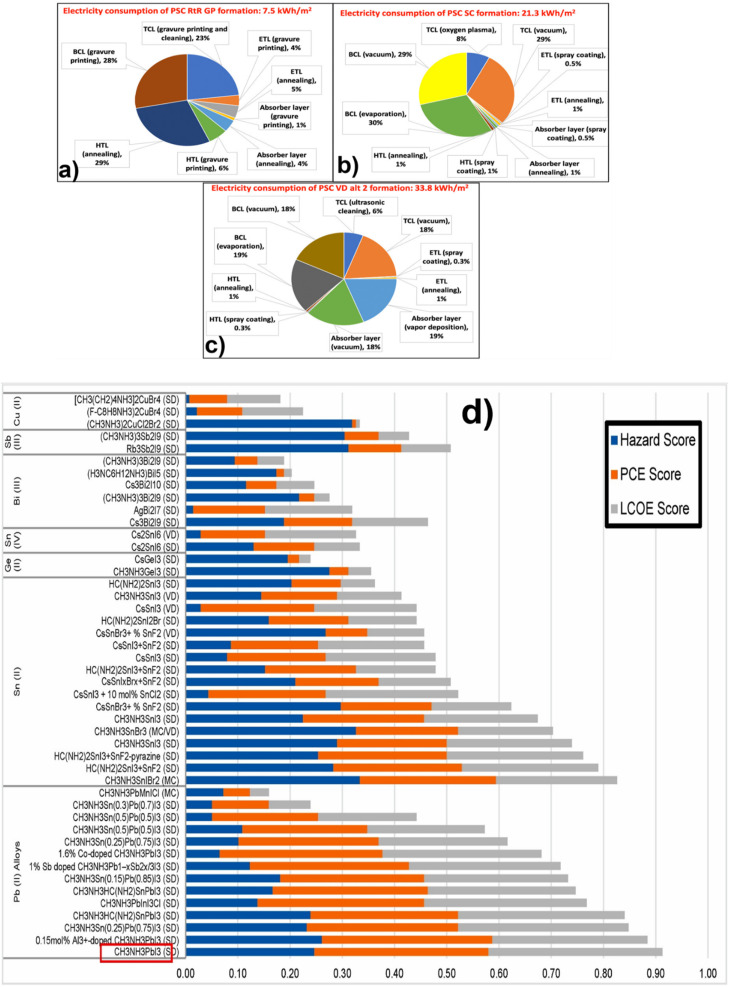
(a–c) Electricity consumption breakdown for the fabrication of a typical perovskite solar cell (b) with roll-to-roll fabrication method, (c) with sheet-to-sheet solution-processed method, (d) with vapor deposition method. Reproduced with permission from ref. 302. (d) Alternative perovskite composition ranking. The closer a perovskite's summed score is to 1, the better its aggregated performance.^333^ SD = Solution deposition, MC = Mechanochemical deposition, VD = Vapor deposition, RtR = Roll-to-roll, GP = Gravure printing, SC = Spray Coating, TCL = Transparent conductive layer, BCL = Back contact layer, ETL = Electron transport layer, HTL = Hole transport layer.

Leccisi *et al.* compared the electricity consumption of perovskite solar cells with FTO/SnO_2_/MAPbI_3_/CuSCN/MoO_*x*_/Al with three different fabrication methods of gravure printing (GP), spray coating (SC), and vapor deposition (VD) (Fig. 16a–c). They studied all the layers including transparent conductive layer (TCL), ETL, perovskite absorber layer, HTL, and back contact layer (BCL). A comparison between these methods revealed that roll-to-roll (R2R) printing with GP method has the lowest environmental impact.^302^ In the spray-coated PSC the deposition of the front and back electrode, assumed to be deposited *via* vacuum-based techniques, contribute about 88% to the total required energy. R2R fabrication, which assumes that also the electrodes are deposited *via* solution processing, is expected to lower the energy consumption of manufacturing substantially from 21 kW h m^−2^ to 7.5 kW h m^−2^. The latter is around 22% of that of vapor deposited devices (33.8 kW h m^−2^) according to the same study. It is apparent that vapor deposition increases energy consumption. The difference is much greater compared to studies by Serrano-Lujan *et al.*,^196^ Espinosa *et al.*,^329^ and Celik *et al.*^324^ for which the estimated lowering in direct electricity inputs (kW h m^−2^) for thermally evaporated cell manufacturing to solution processing were around 26%, 26%, 8% respectively.^331^ Such a notable dissimilarity needs further detailed investigation. Use of flexible substrate also reduces the total mass of devices up to 97%.^319^ Finally, sometimes overlooked in studies, due to stability issues, PSCs require encapsulants which can be glass for rigid modules to more complex multilayer barriers for flexible cells.^332^ Encapsulation can contribute between 37% and 61% of the total energy footprint^302^ which is a significant number. Of course, the more stable the intrinsic material of the solar cells, then less sophisticated solutions for encapsulation required, especially for flexible devices.^332^

Alberola-Borra *et al.* investigated HTM-free PSCs with screen printed mesoporous TiO_2_/ZrO_2_ ETL and carbon electrode to reach a conclusion about pre-industrial PSCs.^336^ They measured different categories, namely abiotic depletion (ADP), abiotic depletion-fossil fuels (ADPF), GWP, ozone layer depletion (ODP), photochemical oxidation (POP), acidification (AP), cumulative energy demand (CED), human toxicity-cancer effects (HTC), human toxicity-non cancer effects (HTNC), and fresh water ecotoxicity (FET). According to their research, in all categories except for POP the perovskite layer has a significant percentage of impact together with other layers. The preparation and annealing of the perovskite layer was responsible for the high impact of this layer rather than its Pb content. Thus, the optimization of the deposition of the perovskite film could be an effective route for reducing environmental impact of PSCs.

Using the ‘USEtox’ method, in 2021 Vidal *et al.* assessed human health and environmental impacts of 8 solvents used in the deposition of the PSCs.^113^ According to their estimations, to produce 1 GW power from PSCs with 15% of efficiency, 3500 litres of solvents are required. They concluded that use of DMSO has lowest human health toxicity and environmental impact expressed in DALYs per kg of a substance emitted for the scenario of emission to urban air among DMF, DMAc, NMP, THF, DMEU, GBL, and DMPU solvents.

To compare PVMs to other PV technologies, a practical approach is to estimate the energy payback time (EPBT), which is the time that it takes solar modules to generate enough energy to surpass the energy required to produce and install them. Solution-processing and low-temperature fabrication lead to lower EPBT of perovskite solar modules than other PV technologies.^239,241,302,324^ The estimated EPBT of PVMs varies from study to study depending on factors such as efficiency and lifespan. One study revealed EPBT of 0.3–0.4 years for single-junction perovskite with 19.3% PCE and all-perovskite tandem solar cells with a PCE of 28%.^302^ This EPBT is about 0.9 years for sc-Si PV with 20% PCE and 0.7 years for 2-terminal Si-perovskite tandems with 28% PCE. Taking into account cradle to gate LCA studies (from cradle to exiting the factory gate), PSCs can be considered greener than silicon PVs.^329^ However, when cradle to grave studies are taken into account, because of the shorter lifetime of PVMs (1–5 years), their environmental impacts are estimated to be higher than current commercial PV technologies that last more than 20 years.^239,241,324^ Vidal *et al.* collected the global warming potential (GWP) and cumulative energy demand (CED) resulting from different LCA studies of single-junction and tandem PSCs.^334^ GWP represents the equivalent mass of CO_2_ emission in kg unit, and CED is the sum of direct and indirect energy usage during the life cycle of products. For 1 kW power production, the mean values of GWP and CED were 2000 kg CO_2_ eq and 39 000 MJ for single-junction PSCs whereas for tandem were 2942 kg CO_2_, 13 744 MJ.^334^ These mean values are lower than those of other PV technologies, but the range of this data is broad due to the lack of real manufacturing data for the perovskite technology. Llanos *et al.*^333^ and the U.S. National Research Council (NRC) studied 45 perovskite materials and their fabrication processes according to human and ecological hazards, efficiency, and cost analysis to find safer materials and solvents. As shown in Fig. 16d, perovskite materials, including lead-free alternatives, and their deposition technique were ranked 0 to 1.^333^ The scores involve hazards, power conversion efficiency (PCE), and the Levelized cost of electricity (LCOE). If the score is close to 1, it represents the better choice of materials and deposition method. This study concluded that the mechanochemical deposited lead-free CH_3_NH_3_SnIBr_2_ would be the most suitable perovskite material considering the combination of the above factors. Additionally, it states that among all lead-based perovskite materials, CH_3_NH_3_PbI_3_ (SD) is the better option. This suggests that these specific materials have been found to have the highest performance in terms of hazard, PCE, and LCOE among their respective groups.

To conclude, LCA studies demonstrate that apart from material selection, the key to reducing the environmental footprint of PVMs is not only increasing the PCE but also simplifying the fabrication process and obtaining long-term stability.

## 8. Conclusion and outlook

In this review, we started by discussing solvents that can be considered “green”. Selection guides such as GSK and CHEM21 helped categorize solvents. Together with Safety (S), Health (H), Environment (E) scores, it is clear that the worst solvents for the perovskite layer are DMF, DMAc, NMP whereas DMSO and ACN fare better, with IPA and water the greenest alternatives already used for some precursors (Pb (NO_3_)_2_). For antisolvents, ethyl acetate and anisole are green solvents that have been used to engineer high quality polycrystalline films with cell efficiencies surpassing the 20% efficiency threshold. LCA studies using the ‘USEtox’ method concluded that the use of DMSO has the less human health toxicity and environmental impact. Further discussed is a new set of solvents called neoteric solvents such as ionic liquids (ILs), methylammonium carboxylates (such as formate, acetate, or propionate) in the preparation of the perovskite layer. In this case, liquid salts don't act as solvents as the methylammonium cations are also incorporated in the final perovskite. ILs are considered promising alternative green solvents to traditional polar aprotic solvents to produce efficient and stable PSCs (*e.g.* cells with FAPbI_3_ combined with methylammonium formate have reached PCEs of 24.1%). Additionally, perovskite films prepared by ILs don't need antisolvent treatments, and films deposited in ambient environments are more durable making the manufacturing of PSCs simpler in the industries.

Solvents are important aspects, but also the perovskite composition must be considered for environmental and safety concerns, trying to either deal with the Pb content, *e.g.*, by lead sequestration/encapsulation strategies, or by synthesizing lead-free perovskites. Bi and Sb are less-toxic alternatives to Pb. However, less toxicity alone does not make a perovskite material superior to lead-based ones. Due to its abundant supply, low price, and global production, Pb has a competitive advantage over Sb and Bi when it comes to production. The efforts to fabricate lead-free perovskites solar cells have proven that no best alternative material can replace the lead completely at the moment. In terms of abundance, cost, global production, global warming potential, and efficiency, Pb is currently the right choice of material. The international community here must continue to work on the containment and replacement of Pb as well as liaise with regulatory bodies to understand how/if Pb-containing perovskite solar panels are able to be deployed for future commercialization.

Among the electron transport layer materials SnO_2_ with a record efficiency of 25.7% is currently considered the best option for low-temperature processing, for compatibility with flexible substrates and for being cast from water-based inks. The choice for hole transport material is less straightforward since most of them are organic with concerns over their stability. Currently, research is going on testing different interface layers with HTLs for stabilization. Inorganic NiO_*x*_ with efficiencies of over 20% have also been proven to be a good HTL. Easy processing Inorganic HTL NiO_*x*_ in p–i–n structures may be a better choice when compared to spiro and PTAA in n–i–p structures. Also, NiO_*x*_ is cast from green solvents like water, IPA, and ethanol.

Currently, many research groups use expensive Au, Ag, and Al top electrodes. Gold electrodes can contribute to more than 50% of prime energy consumption and carbon footprint in the fabrication of perovskite solar modules. Metallic top electrodes such as Cu, Ni, W, and Mo, have been explored, but are still sputtered or evaporated as well as less stable than Au. Carbon-based electrodes are one of the best electrode materials considering both stability and environmental concerns. Solvents and binders used for processing carbon materials like-ethanol, ethyl acetate, terpineol, cellulose, organic acetates, and IPA are non-toxic. Carbon is deposited by solution-processed methods such as screen printing and spray coating, which reduces the embedded energy of perovskite solar modules. For instance, deposition of carbon front contacts by spray coating leads to 35% less process energy consumption than evaporated MoO_*x*_/Al electrode. Further research into this field should close the efficiency gap that still exists compared to devices with Au electrodes. Recent developments in bio-carbons produced from biomass waste such as aloe-vera peel, fallen quince leaves, rice husk, bamboo chopsticks, peanut shell bio-carbon, corn stalk, and phragmites australis, *Eichhornia crassipes*, and soybean dregs further reduces the cost of carbon electrodes.

According to the life cycle assessment of a typical glass/FTO/TiO_2_/spiro-OMeTAD/Au perovskite solar cell architecture, FTO preparation accounts for 28% of device fabrication energy and 44% of embedded material energy. The presence of tin in ITO and FTO presents toxicity and supply concerns. Using aluminum-doped zinc oxide (AZO) as front contact is a prospective replacement with less environmental impact. Glass accounts for around 98% of the total mass of PSCs because of its relatively higher thickness than other layers. To reduce the embedded energy of the substrate, alternatives such as flexible PET, PEN, paper, and cellulose-based substrates can be considered. Plastic based substrates have surpassed the 21% efficiency threshold with exceptional power-to-weight ratios. However flexible technology can only be considered for products which do not require long lifetimes, until the performance and cost of encapsulation and intrinsic materials enable long term use. More research should be funneled for this aim.

## Conflicts of interest

There are no conflicts to declare.

## Supplementary Material
